# Supplementary material for: Proton transfer regulated photocured robust room-temperature phosphorescence from naphthalimide
Source: Nat Commun. 2026 Mar 21;17:4287. doi: 10.1038/s41467-026-70999-8 (PMC13168491; doi:10.1038/s41467-026-70999-8)
Supplement: Supplementary file 1 — Supplementary Information [file 41467_2026_70999_MOESM1_ESM.pdf]

Supplementary Information

Proton Transfer Regulated Photocured Robust Room-Temperature Phosphorescence from Naphthalimide

Aicheng Wang,[1] Haoxuan Wei,[2] Kunquan Lin, [1] Xing Huang,[3] Mingxing Chen,[4] Wentao Bian,[1] Junxiao Wang,[2] Yuzhou Qiao,[1] Bing Fang,[1*] Yuxia Zhao,[3] Jianxiang Yu,[1] Meizhen Yin,[2*] and Yuhua Dai[1*]

[1]Beijing Key Laboratory of Special Elastomer Composite Materials, College of New Materials and Chemical Engineering, Beijing Institute of Petrochemical Technology, Beijing 102617, China

[2]State Key Laboratory of Chemical Resource Engineering, Beijing University of Chemical Technology, Beijing 100029, China

[3]Key Laboratory of Photochemical Conversion and Optoelectronic Materials, Technical Institute of Physics and Chemistry, Chinese Academy of Sciences, Beijing 100190, China

[4]Analytical Instrumentation Center of Peking, Peking University, Beijing, China

* Email: fangbing@bipt.edu.cn; yinmz@mail.buct.edu.cn; daiyuhua@bipt.edu.cn

Supplementary information

Chemicals and Materials

All chemicals or starting materials were acquired from commercial suppliers and employed directly without additional purification unless otherwise noted.

1,8-Naphthalic anhydride (98 %) and N, N-dimethylethylenediamine (98 %) were purchased from Innochem. Radical scavenger 5,5dimethyl-1-pyrroline N-oxide (DMPO, 97%) was purchased from Aladdin (Shanghai, China). Organic reagents, including ethanol (99.5 %), methanol (99.5 %), N, N-dimethylacetamide (99.5 %), tetrahydrofuran (99.5 %), n-hexane (99.5 %) were purchased from Beijing Chemical Works; methyl tert-butyl ether (99.5 %) from Innochem; and dichloromethane (99.5 %) from Aladdin. The monomer Acrylamide (99.0%), acrylic acid (99.0%) were purchased from Innochem (Beijing, China); methyl acrylate (>99.0%), methyl methacrylate (>99.5%), styrene (≥99.0%), 2-Hydroxyethyl acrylate (96%) were purchased from Aladdin. Poly (vinyl alcohol) (average Mw ~205,000) was purchased from Macklin. Rhodamine B (RhB) was obtained from Aladdin. Acrylated Aliphatic Urethane were purchased from Chengdu fourth city new material company. Deionized water was produced using a ultrapure water system (Volvo Environmental Protection (Shenzhen) Co., Ltd.). UV curing lamp (photocuring wavelength =365 nm, optical density=300mW/cm^2) was purchased from Youwei Gu Lighting Electric Appliance Factory, Guzhen Town, Zhongshan City. All silicone molds, cotton thread, masks and other glass materials are customized for Taobao (Alibaba, Hangzhou, China) stores.

Characterization

^{1}H NMR and ^{13}C NMR spectra were recorded on a Bruker 400 (400 MHz) spectrometer at room temperature. Fluorescence studies were performed on a fluorescence spectrophotometer (FS-5). UV-vis spectra were measured on a spectrometer (Shimadzu 2600, Japan). Delayed emission spectra and lifetime decay curves are recorded by the FLS980 Photoluminescence Spectrometer (Edinburgh Instruments, Livingston, UK) equipped with microsecond lamp. Electron spin resonance (ESR, Bruker A300, Germany) was employed to monitor light-induced radicals. The spectrometer was operated at 9.85 GHz (X-band) with a center field of 3 510 G, a sweep width of 100 G, a modulation frequency of 100 kHz, and a modulation amplitude of 1.0 G; microwave power was set to 19.7 mW, time constant to 10.24 ms, and conversion time to 45 ms. DMPO served as the spin-trapping agent. First-derivative spectra were recorded at room temperature with a single 46-s field sweep (1024 points). The measurements were performed on the prepolymer mixture before and immediately after illumination to quantify the generated radical species.

The photopolymerization kinetics of monomers was studied by real-time FTIR (Nicolet IS20). The C=C double bond conversion (DBC) of P-AA-AM was calculated according to Equation 1 by measuring the area changing of the C=C characteristic absorption peak at 6100-6250 cm^{-1}. The rate of monomer polymerization can be obtained by deriving the DBC.

$$C_{C=C} = \frac{A_0 - A_t}{A_0} \times 100\% \qquad (1)$$

where A_0 is the area at the beginning, A_t is the area at time t.

All photos and videos were taken using a mobile phone.

Calculation of the photophysical parameters

The absolute photoluminescence quantum yields (Φ_{PL}) were obtained on Edinburgh FLS980 fluorescence spectrophotometer equipped with an integrating sphere under ambient conditions. The phosphorescent quantum yield (Φ_{Phos}) was calculated by the following equation:

$$\Phi_{Phos}=\frac{S_2}{S_1+S_2}\times\Phi_{PL}$$

where S1 is the integrated area of the fast-decay (fluorescence) segment in the time-resolved spectrum, and S2 is the integrated area of the slow-decay (phosphorescence) segment; the sum of S1 and S2 represents the total integrated area of the entire time-resolved spectrum.

The fluorescence radiative rate constant (k_r^{Fluo}), fluorescence non-radiative rate constant (k_{nr}^{Fluo}), ISC rate constant (k_{ISC}), phosphorescence radiative rate constant (k_r^{Phos}), and phosphorescence non-radiative rate constant (k_{nr}^{Phos}) were calculated according to the following equations:

$$k_r^{Fluo}=\frac{\emptyset_{Fluo}}{\tau_{Fluo}}$$

$$k_{nr}^{Fluo}=\frac{1-\emptyset_{Fluo}-\emptyset_{Phos}}{\tau_{Fluo}}$$

$$k_{ISC}=\frac{\emptyset_{Phos}}{\tau_{Fluo}}$$

$$k_r^{Phos}=\frac{\emptyset_{Phos}}{\tau_{Phos}}$$

$$k_{nr}^{Phos}=\frac{1-\emptyset_{Fluo}-\emptyset_{Phos}}{\tau_{Phos}}$$

The energy transfer efficiency (Φ_{FRET}) between the energy donor and acceptor calculated from the following equation:

$$\Phi_{FRET} = \frac{\tau_D - \tau_A}{\tau_D} \times 100\ \%$$

Where τ_A and τ_D represent the lifetime of the donor in the presence of an acceptor and without the acceptor, respectively.

Simulation methods

All molecular conformations were optimized and vibrational frequency analyses were performed via the Gaussian 09 program at the B3LYP/6-31G(d,p) level of theory. Excited-state calculations were carried out using the ORCA 4.2.0 program at the PBE0/def2-SV(P) level. Analysis of the electrostatic potential surfaces (ESPs), independent gradient model based on Hirshfeld partition (IGMH) analysis, and natural transition orbital (NTO) analysis for the singlet (S_1) and triplet (T_1) excited states were completed with the Multiwfn_3.8_dev program[1], and the corresponding plots were generated using the VMD 1.9.3 program.

Supplementary methods

Photocuring of P-AA

A mixture containing 400 μL of acrylic acid (5.83 mmol), and 1 mg of NDIAM was heated to 60 °C until it formed a homogeneous solution. Subsequently, the solution was subjected to photocuring under a 365 nm UV lamp source with an intensity of 300 mW cm^{-2} for 300 seconds.

Photocuring of P-St / P-MA / P-MMA / P-HEA

1 mg of NDIAM was dissolved in 120 ul of DMF, and then 500 mg of

monomers (St-4.80 mmol, MA-5.81 mmol, MMA-4.99 mmol, HEA-4.31 mmol) were added, respectively, followed by ultrasonication for 30 min until a homogeneous solution was formed. Subsequently, the solution was subjected to photocuring under a 365 nm UV lamp source with an intensity of 300 mW cm^{-2} for 500 seconds.

Photocuring of P-AA-St / P-AA-MA / P-AA-MMA / P-AA-HEA

1 mg of NDIAM was dissolved in 400 uL of AA (5.83 mmol), and then 500 mg of monomers (St-4.80 mmol, MA-5.81 mmol, MMA-4.99 mmol, HEA-4.31 mmol) were added, respectively, followed by ultrasonication for 30 min until a homogeneous solution was formed. Subsequently, the solution was subjected to photocuring under a 365 nm UV lamp source with an intensity of 300 mW cm^{-2} for 300 seconds.

Preparation of PVA solution and various NDIAM doped films.

Accurately weigh 10 g of polyvinyl alcohol (PVA) in a 250 mL beaker, add 100 mL of deionized water, stir and heat at 80 °C for 3 h until it is completely dissolved, and obtain a homogeneous PVA solution with a mass concentration of 100 mg/mL.

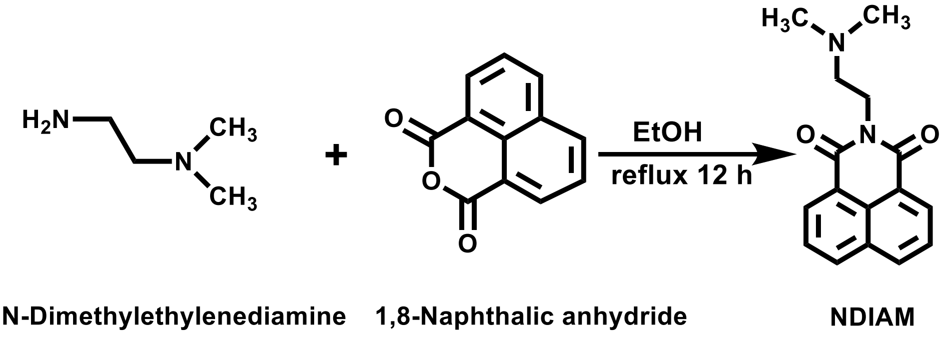

Supplementary Fig. 1 The synthesis route of NDIAM.

Synthesis of NDIAM

1, 8-naphthalenedicarboxylic anhydride (792 mg, 4 mmol) was added in portions to a solution of 352 mg (4 mmol) of N^1, N^1-dimethylethane-1,2-diamine in 10 mL of EtOH and the mixture was heated to reflux during 12 h. The mixture was filtered under vacuum and the filtrate was evaporated under reduced pressure. The residue was purified by chromatography (DCM : MeOH = 8:1) to afford NDIAM as a faint yellow powder in a yield of 85%. ^1H NMR (400 MHz, $CDCl_3$) δ 8.63 (d, J = 7.1 Hz, 2H), 8.23 (d, J = 8.3 Hz, 2H), 7.80 - 7.75 (m, 2H), 4.36 (t, J = 7.1 Hz, 2H), 2.68 (t, J = 7.1 Hz, 2H), 2.38 (s, 6H), 1.71 (s, 2H). ^{13}C NMR (101 MHz, $CDCl_3$) δ 164.24, 133.91, 131.59, 131.24, 128.21, 126.92, 122.69, 57.00, 45.77, 38.18. ESI-TOF: $C_{16}H_{16}N_2O_2$, *m/z* calcd for $[M+H]^+$, 269.12; found, 269.12.

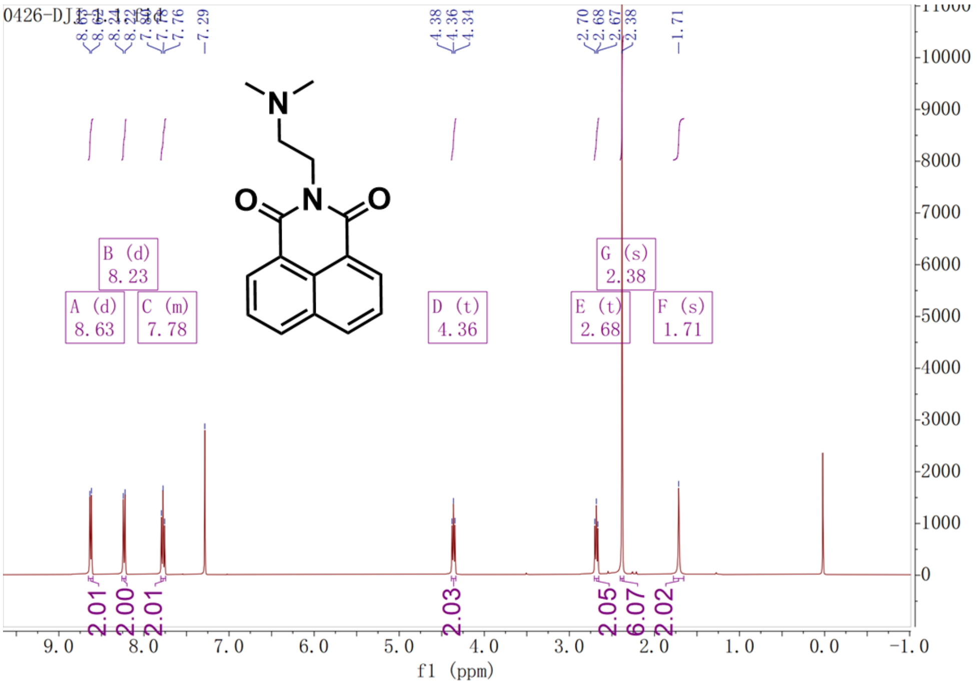

Supplementary Fig. 2 ^1H NMR (400 MHz, $CDCl_3$) of NDIAM.

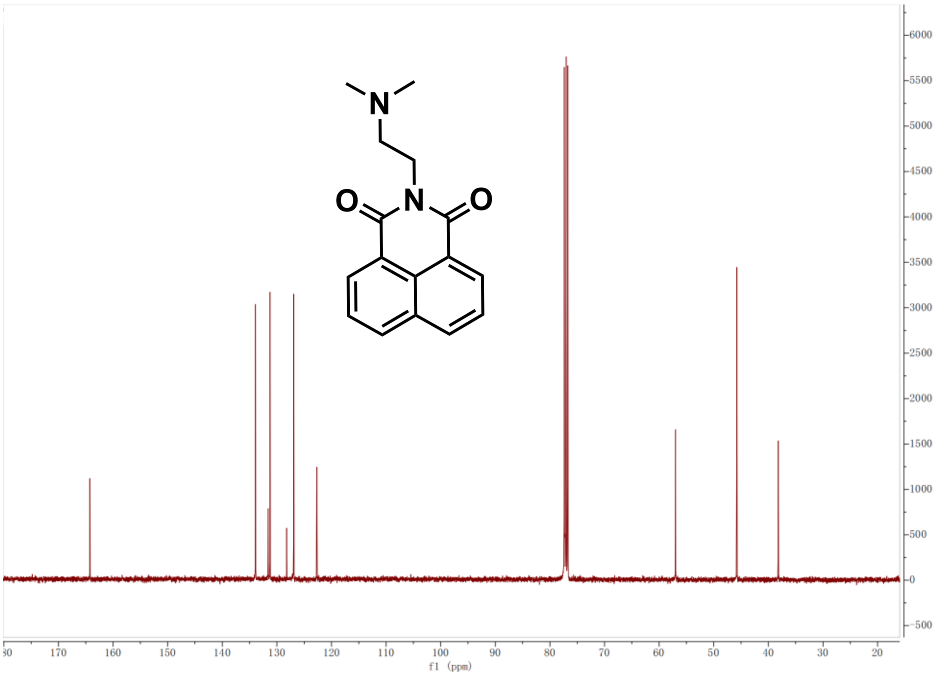

Supplementary Fig. 3 ^{13}C NMR (101 MHz, $CDCl_3$) of NDIAM.

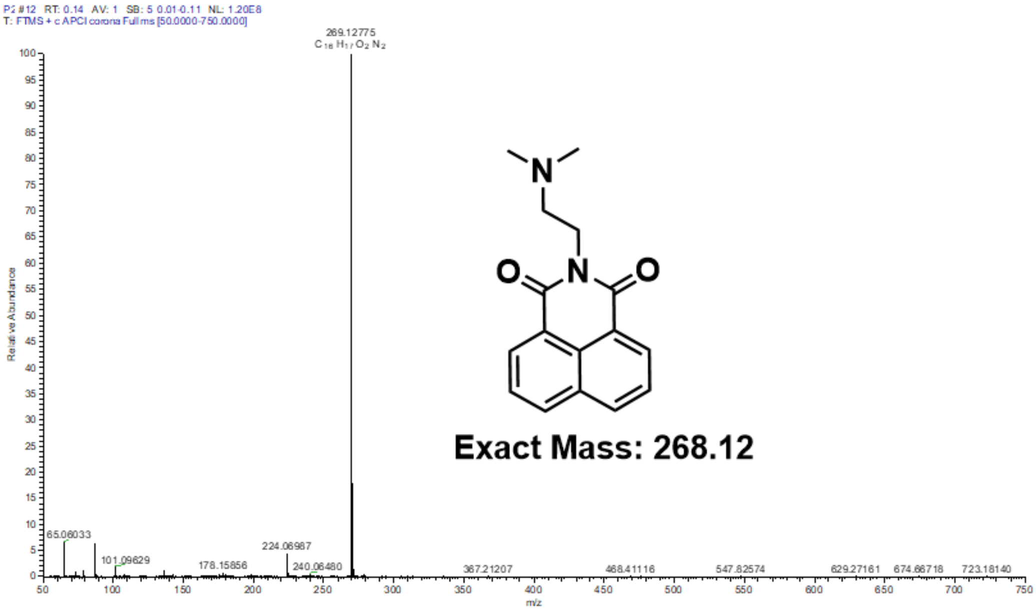

Supplementary Fig. 4 ESI-TOF of NDIAM.

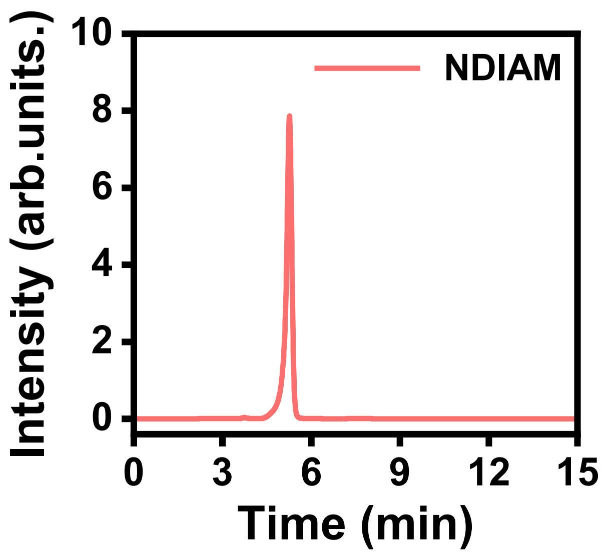

Supplementary Fig. 5 HPLC spectrum and its quantitative analysis result of NDIAM in methanol elution for 15 min. Elution rate: 0.6 mL min^{-1}.

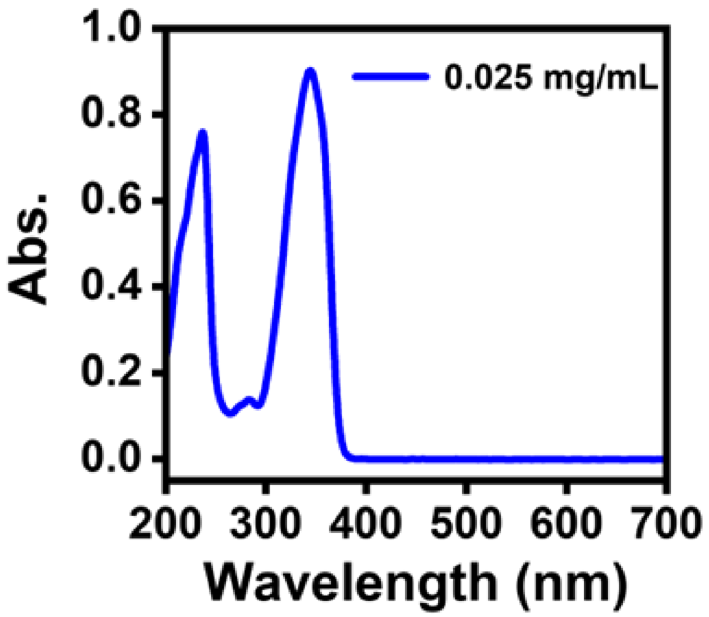

Supplementary Fig. 6 UV-vis spectrum of NDIAM in AA (0.025 mg/mL).

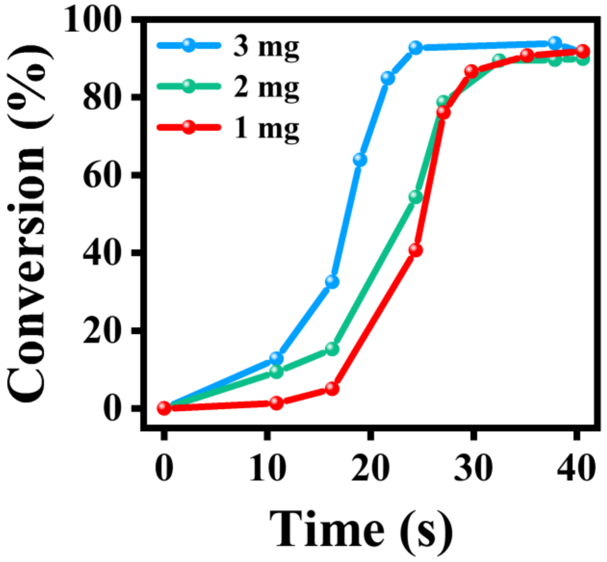

Supplementary Fig. 7 The double bond conversion rate of P-AA-AM prepared with different mass NDIAM (1, 2, and 3 mg), 400 μL AA and 500 mg AM.

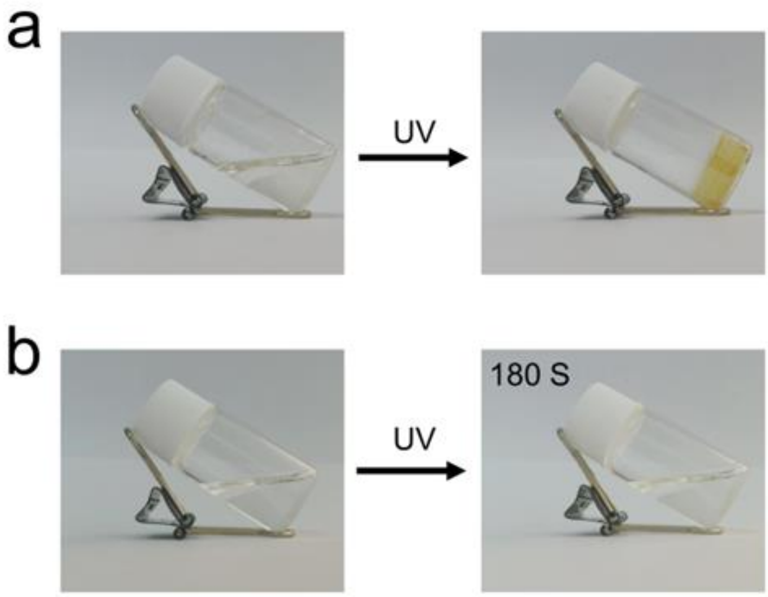

Supplementary Fig. 8 Digital image after P-AA-AM (NDIAM: 3.7 μM) photocuring. a) without adding DMPO; b) after adding DMPO (300 μM).

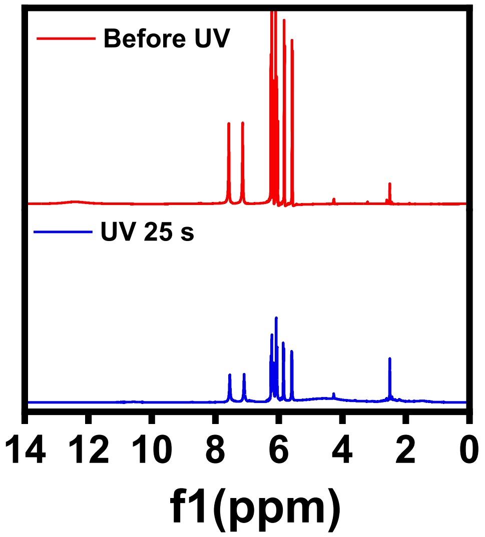

Supplementary Fig. 9 ^{1}H NMR (400 MHz) of NDIAM (3 mg) and mixtures of AA (400 μL) and AM (500 mg) in DMSO-d6 a) before and b) after UV irradiation for 25 s.

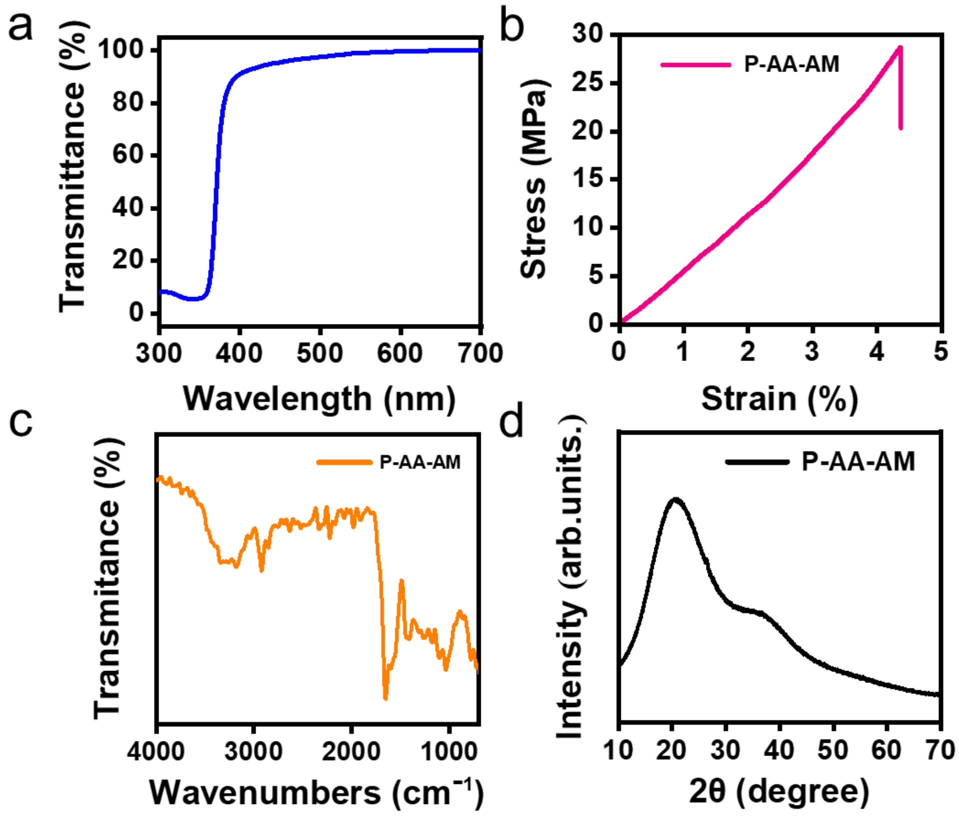

Supplementary Fig. 10 a) UV-vis transmittance spectrum; b) mechanical strength; c) FTIR spectrum; d) X-ray diffraction pattern of P-AA-AM.

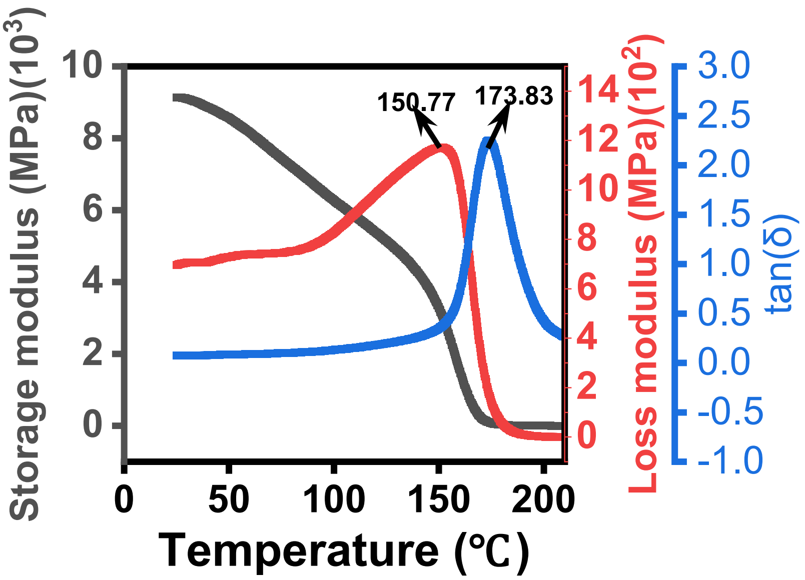

Supplementary Fig. 11 DMA curves of P-AA-AM: storage modulus, loss modulus, and tan(δ) versus temperature.

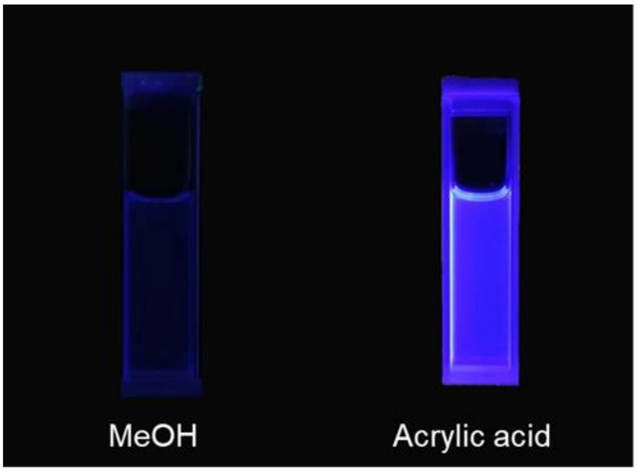

Supplementary Fig. 12 After NDIAM was dissolved in MeOH (left) and AA (right), respectively, the fluorescence digital images were taken under 365 nm UV light.

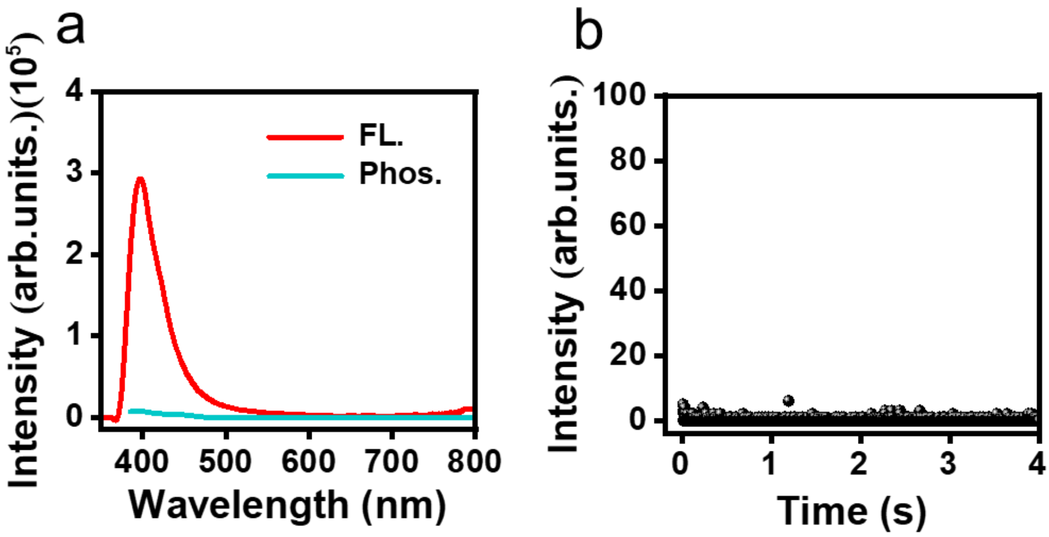

Supplementary Fig. 13 RTP emission of NDIAM in the mixed solution of AA and AM, a) prompt (red line) and delayed (green line) emission spectra of NDIAM dissolved in the mixed solution; b) Phosphorescence lifetime of NDIAM in mixed solution (no signal was detected). $\lambda_{exc.}$ = 365 nm, $\lambda_{collected}$ = 550 nm, delay time = 1 ms.

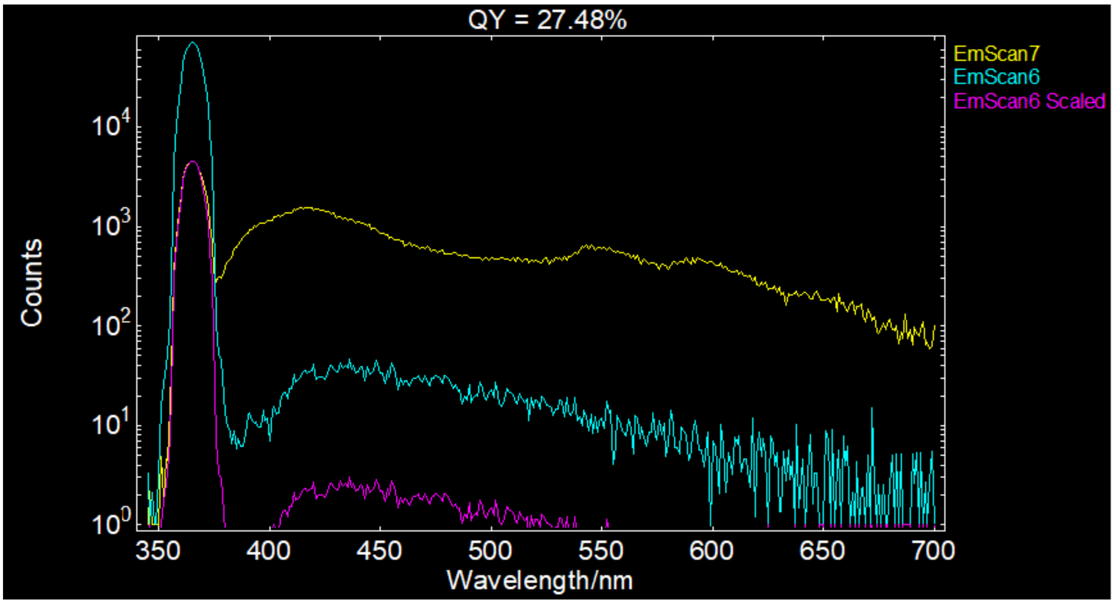

Supplementary Fig. 14 Photoluminescence quantum yield of a P-AA-AM.

The phosphorescent quantum yield ($\Phi_{Phos.}$) was calculated by the following

equation according to the method reported in previous literature:

$$\Phi_{Phos}=\frac{S_2}{S_1+S_2}\times\Phi_{PL}$$

where S_1 is the integrated area of the fast-decay (fluorescence) segment in the time-resolved spectrum, and S_2 is the integrated area of the slow-decay (phosphorescence) segment; the sum of S_1 and S_2 represents the total integrated area of the entire time-resolved spectrum. $\Phi_{Phos.}$ = 17.83%, $\Phi_{Fluo.}$ = 9.65%.

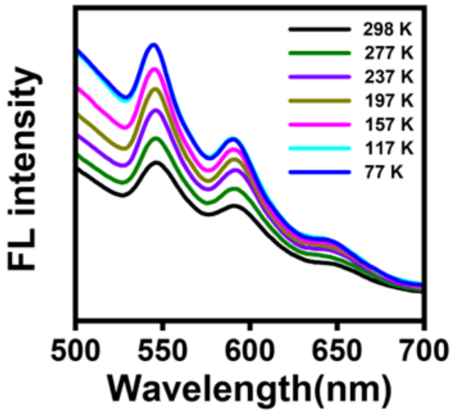

Supplementary Fig. 15 Temperature dependent fluorescence (FL) spectra of P-AA-AM at 77 K, 117 K, 147 K, 197 K, 237K, 277K, 298 K.

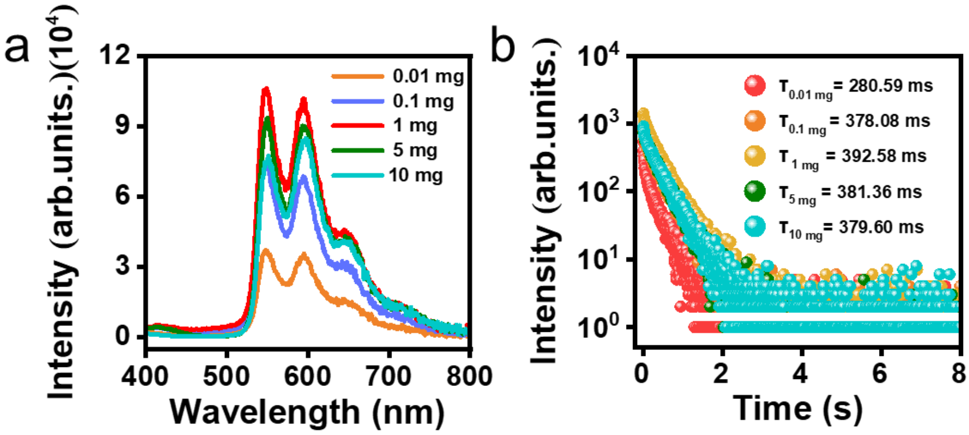

Supplementary Fig. 16 The influence of mass of NDIAM on phosphorescent intensity a) and lifetime b).

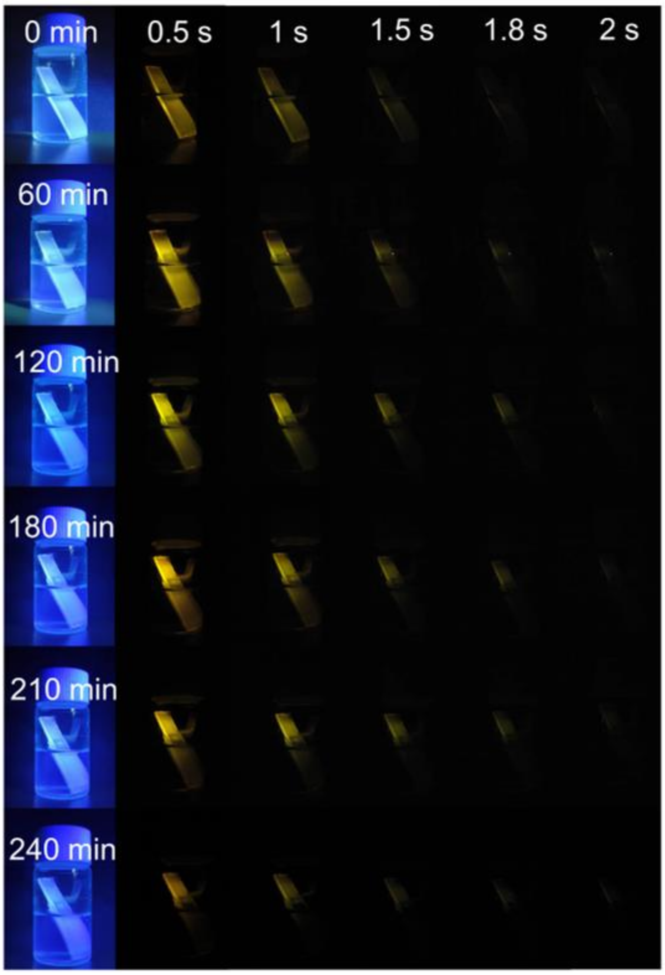

Supplementary Fig. 17 Afterglow images of P-AA-AM immersed in water for different times (Sample length 3.5 cm, width 0.5 cm, thickness 0.4 mm).

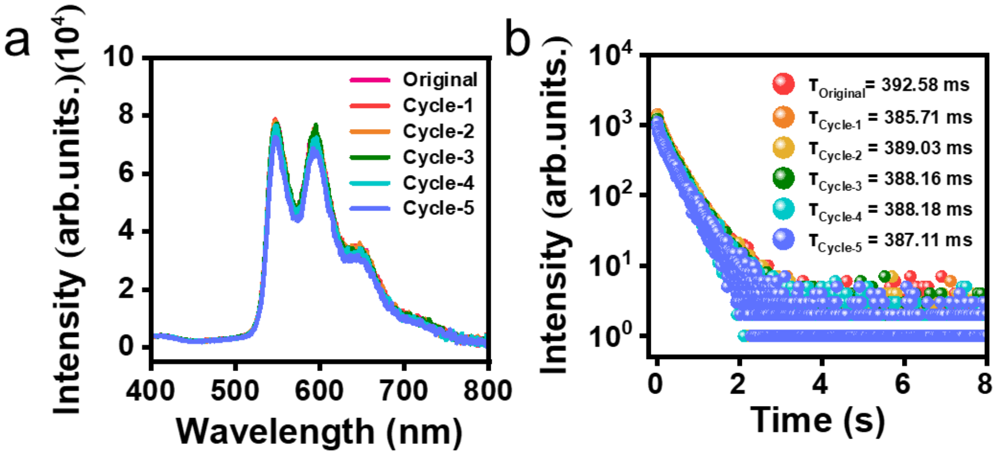

Supplementary Fig. 18 The RTP intensity a) and lifetime b) of P-AA-AM after removing the water.

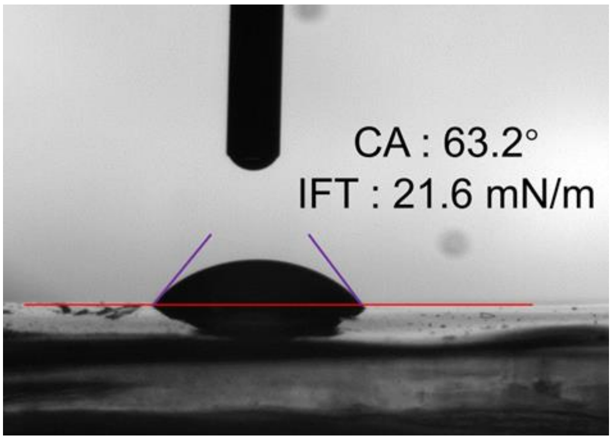

Supplementary Fig. 19 The water contact angle of P-AA-AM.

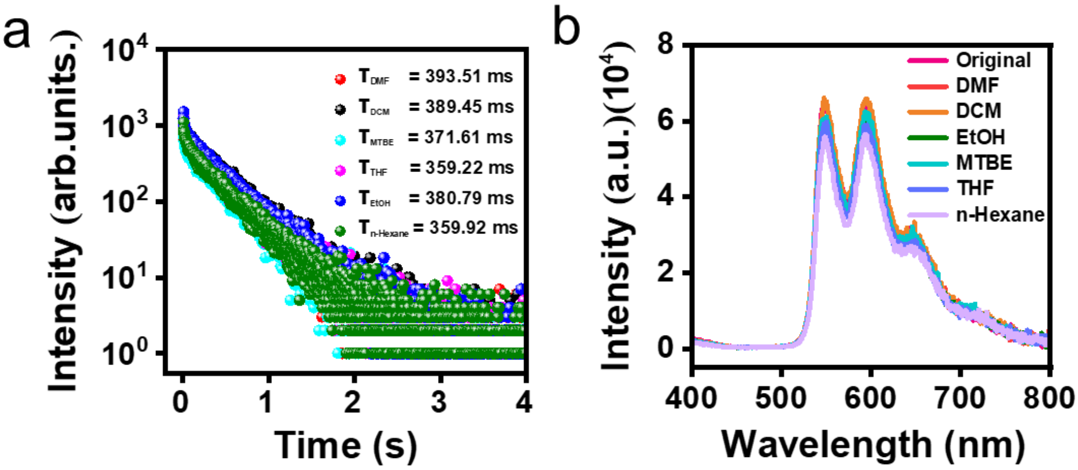

Supplementary Fig. 20 a) RTP lifetime and b) intensity of P-AA-AM after soaking in different organic solvents.

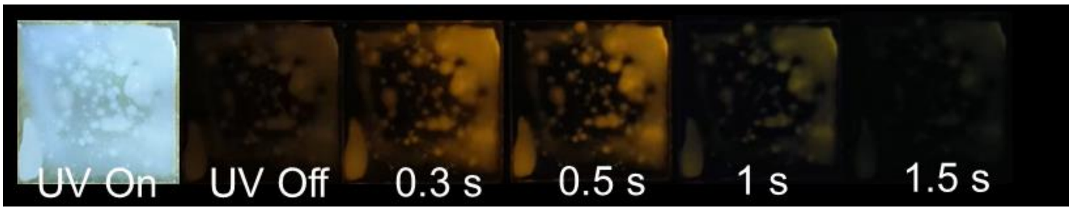

Supplementary Fig. 21 The afterglow of P-AA film.

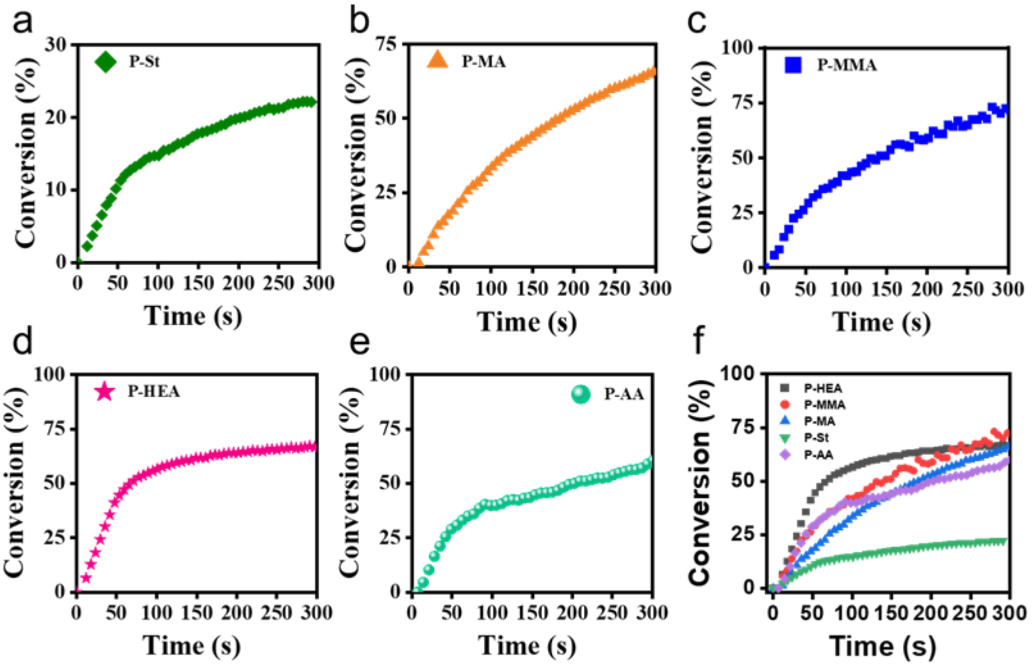

Supplementary Fig. 22 The double bond conversion of a) P-St, b) P-MA, c) P-MMA, d) P-HEA, e) P-AA and f) The summary of double bond conversion of various monomers under 365 nm UV irradiation different time.

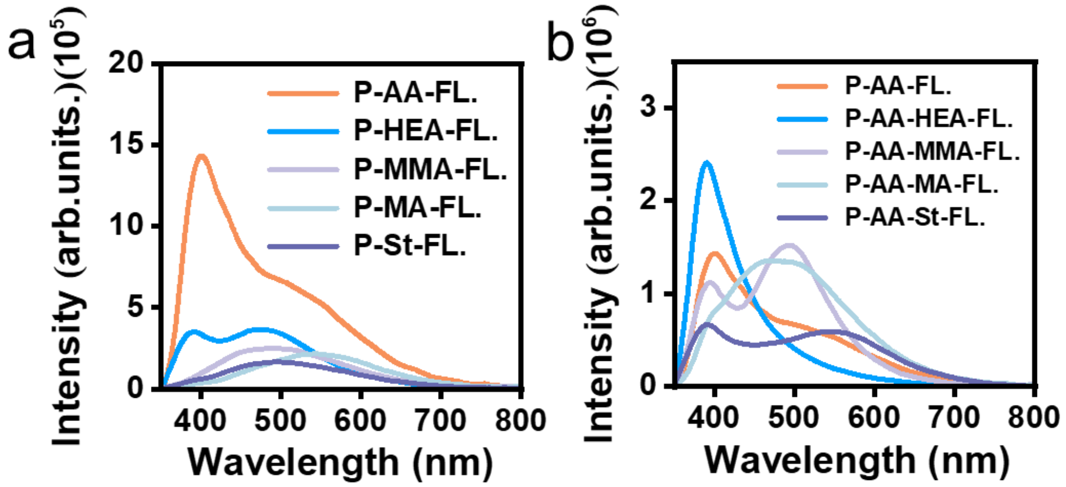

Supplementary Fig. 23 Fluorescence (FL) spectra of photocuring materials by a) polymerizing different monomers or b) copolymerizing different monomers with AA.

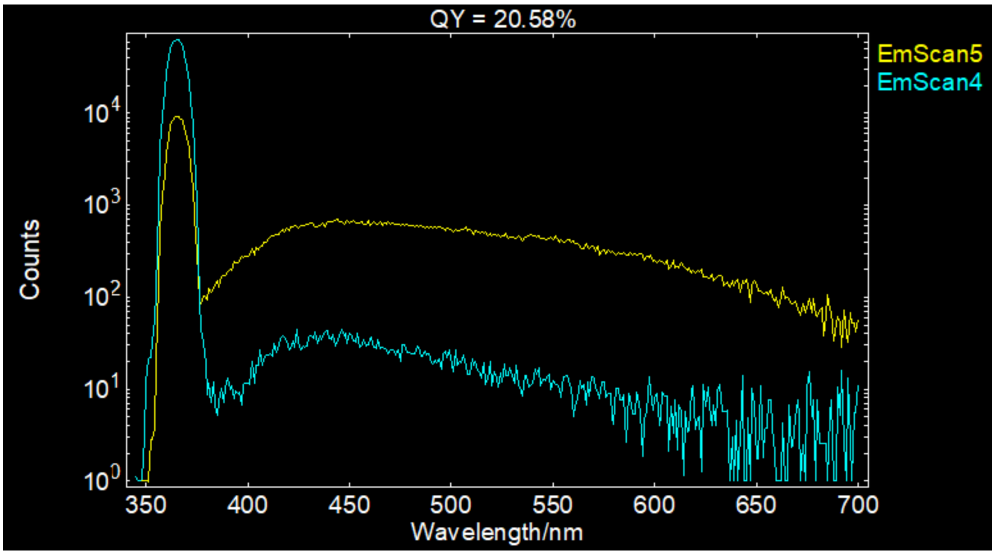

Supplementary Fig. 24 Photoluminescence quantum yield of a P-AA.

The phosphorescent quantum yield ($\Phi_{Phos.}$) was calculated by the following equation according to the method reported in previous literature:

$$\Phi_{Phos}=\frac{S_2}{S_1+S_2}\times\Phi_{PL}$$

where S_1 is the integrated area of the fast-decay (fluorescence) segment in the time-resolved spectrum, and S_2 is the integrated area of the slow-decay (phosphorescence) segment; the sum of S_1 and S_2 represents the total integrated area of the entire time-resolved spectrum. $\Phi_{Phos.}$ = 12.46%, $\Phi_{Fluo.}$ = 8.12%.

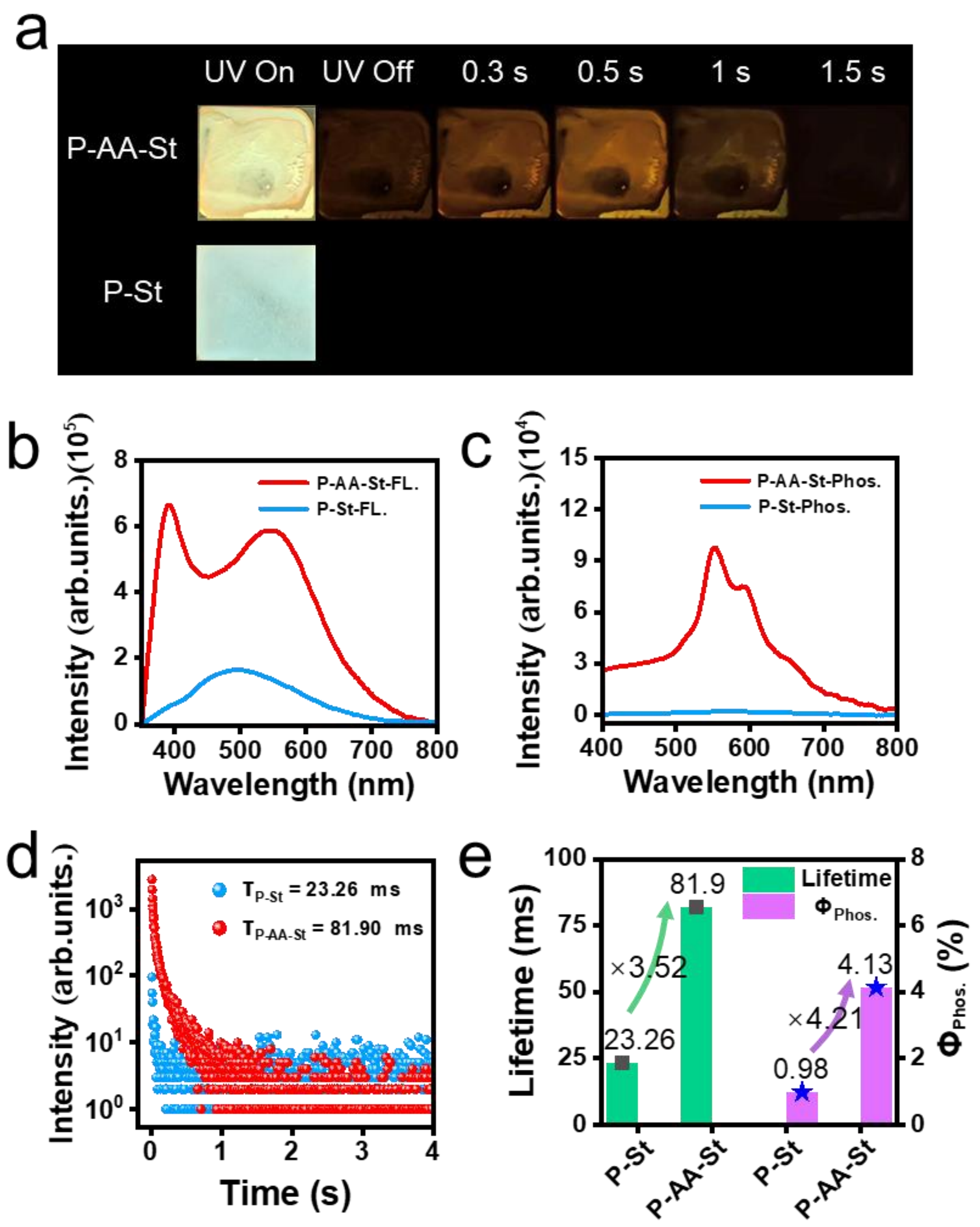

Supplementary Fig. 25 a) Photographs of P-AA-St and P-St under and after ceasing 365 nm UV light irradiation; b) The prompt and c) delayed spectra of P-AA-St and P-St; d) Phosphorescence lifetimes of P-AA-St and P-St; e) Phosphorescence lifetimes and quantum yield of P-AA-St and P-St (single measurement, λ_{ex} = 365 nm).

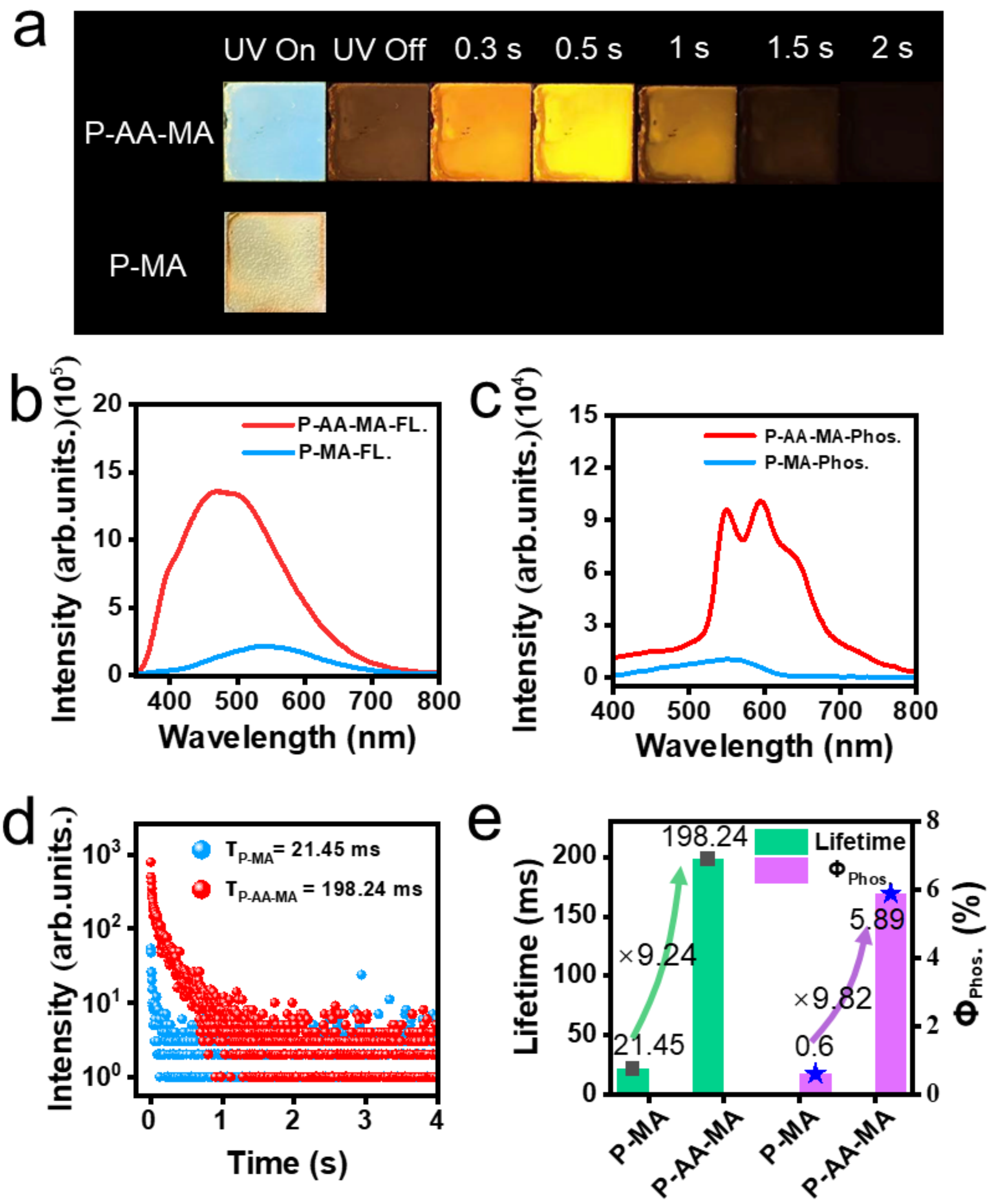

Supplementary Fig. 26 a) Photographs of P-AA-MA and P-MA under and after ceasing 365 nm UV light irradiation; b) The prompt and c) delayed spectra of P-AA-MA and P-MA; d) Phosphorescence lifetimes of P-AA-MA and P-MA; e) Phosphorescence lifetimes and quantum yield of P-AA-MA and P-MA (single measurement, λ_{ex} = 365 nm).

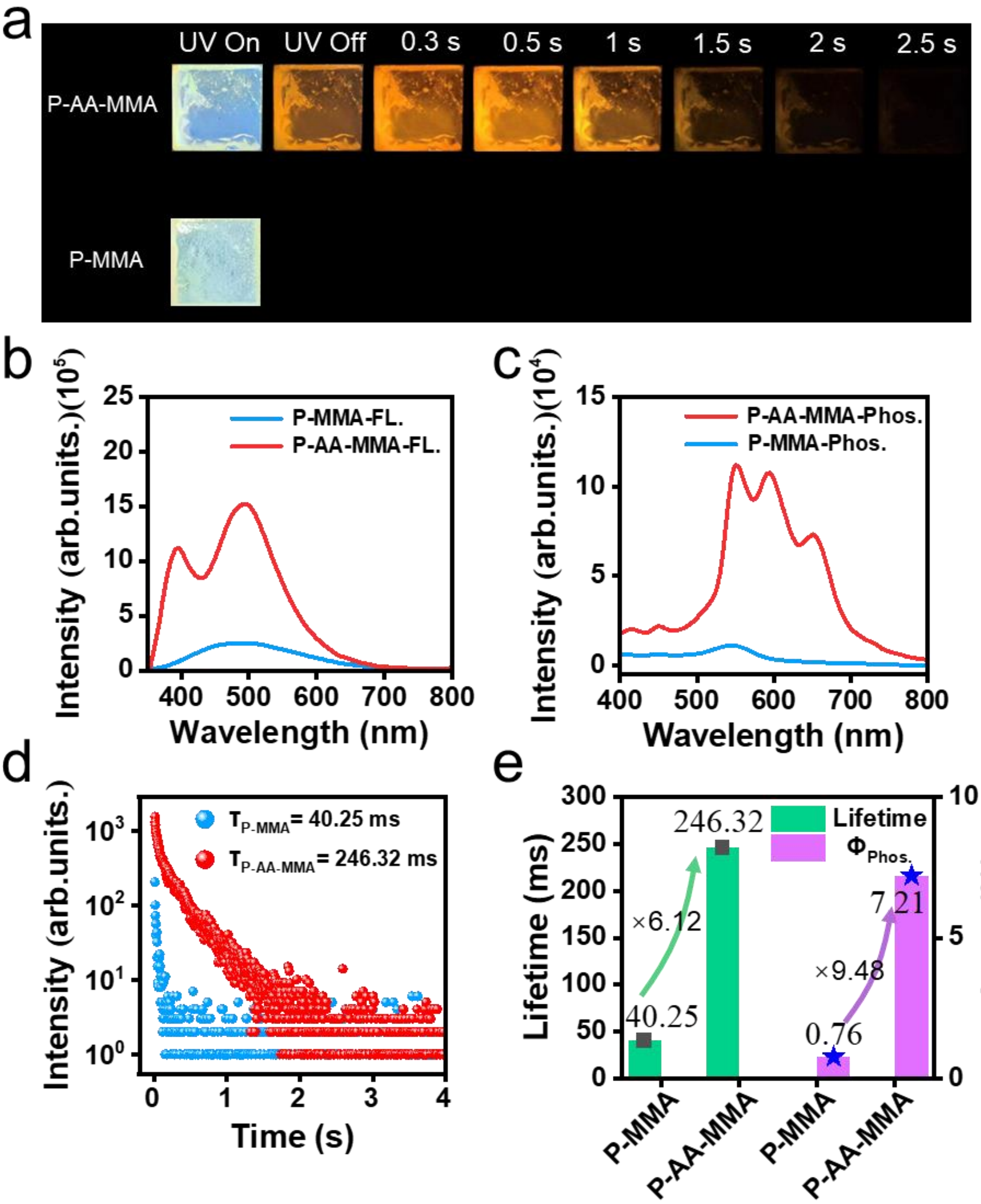

Supplementary Fig. 27 a) Photographs of P-AA-MMA and P-MMA under and after ceasing 365 nm UV light irradiation; b) The prompt and c) delayed spectra of P-AA-MMA and P-MMA; d) Phosphorescence lifetimes of P-AA-MMA and P-MMA; e) Phosphorescence lifetimes and quantum yield of P-AA-MMA and P-MMA (single measurement, λ_{ex} = 365 nm).

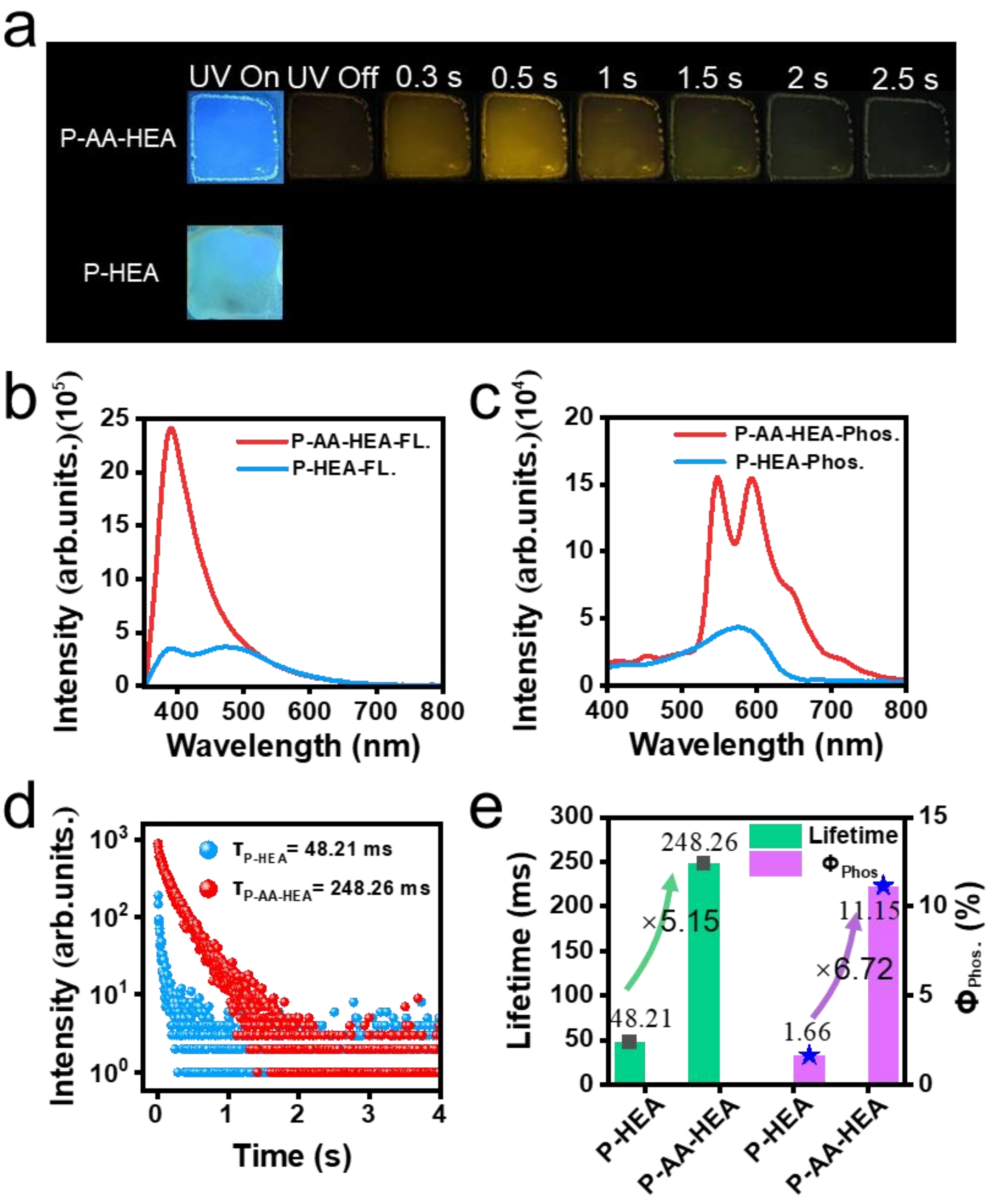

Supplementary Fig. 28 a) Photographs of P-AA-HEA and P-HEA under and after ceasing 365 nm UV light irradiation; b) The prompt and c) delayed spectra of P-AA-HEA and P-HEA; d) Phosphorescence lifetimes of P-AA-HEA and P-HEA; e) Phosphorescence lifetimes and quantum yield of P-AA-HEA and P-HEA (single measurement, λ_{ex} = 365 nm).

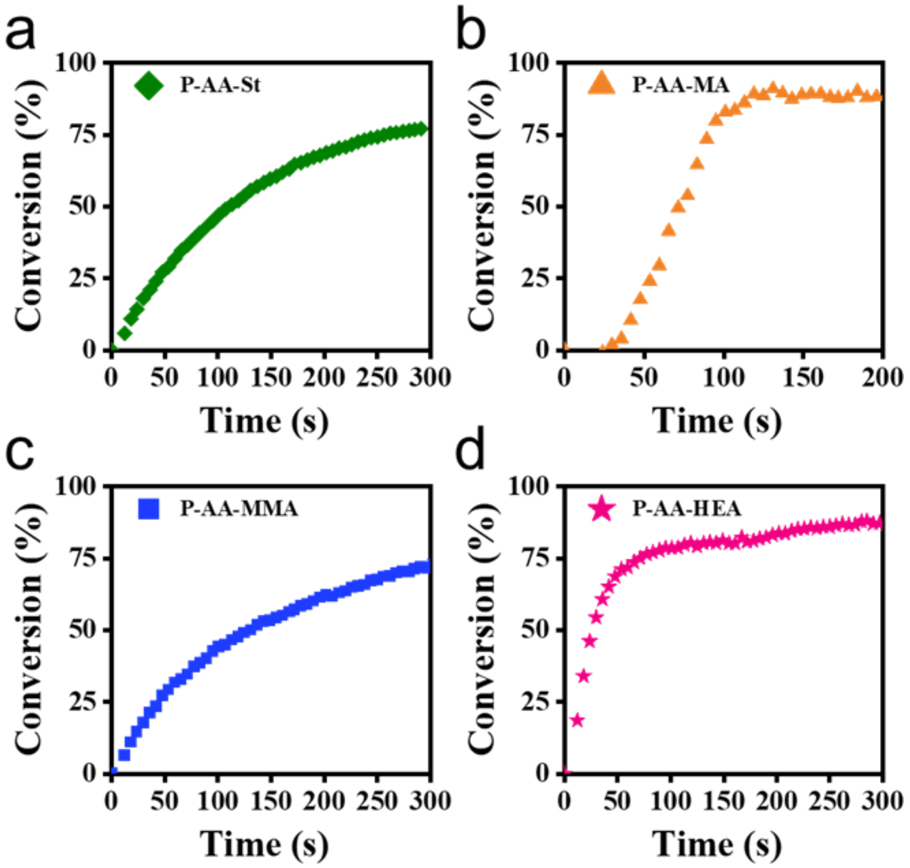

Supplementary Fig. 29 The double bond conversion of a) P-AA-St, b) P-AA-MA, c) P-AA-MMA and d) P-AA-HEA under 365 nm UV irradiation different time.

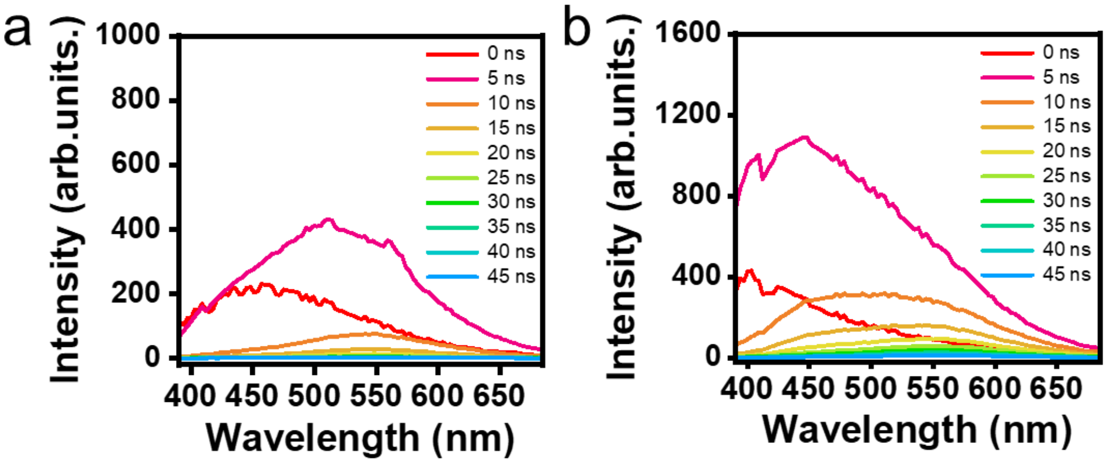

Supplementary Fig. 30 Time-resolved fluorescence spectra of a) P-St and b) P-AA-St.

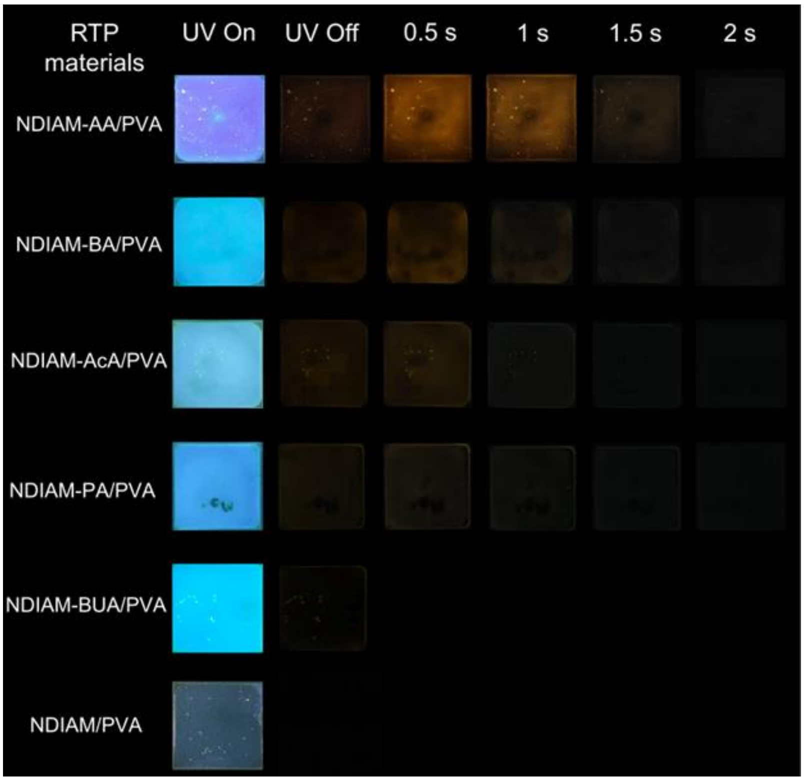

Supplementary Fig. 31 RTP photographs of NDIAM doped PVA films with different acids treatment.

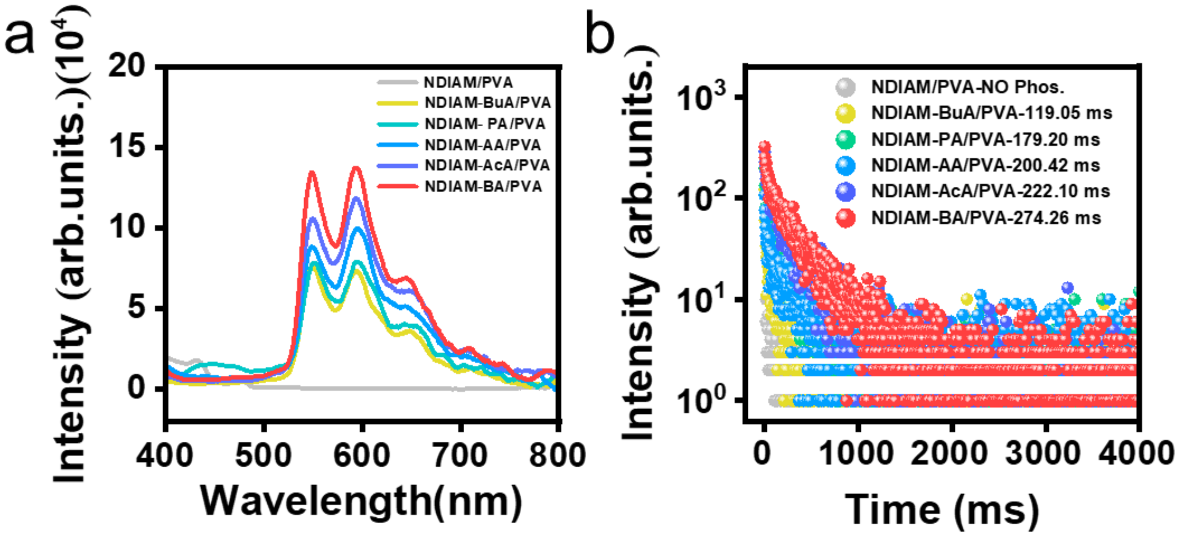

Supplementary Fig. 32 a) Delayed spectra and b) RTP lifetime of NDIAM doped PVA films with different acid treatments.

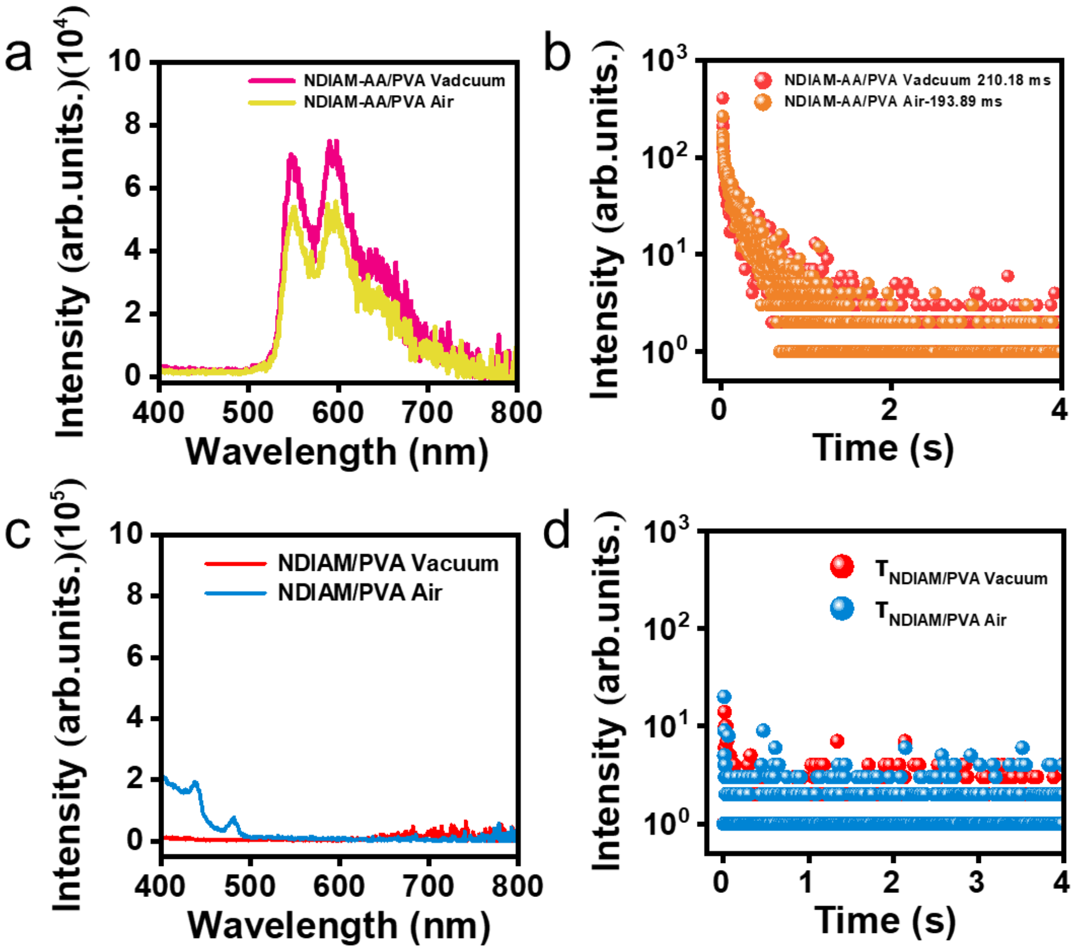

Supplementary Fig. 33 Phosphorescence intensity and Lifetime of NDIAM-AA/PVA a) and b); NDIAM-PVA c) and d) under vacuum and air condition.

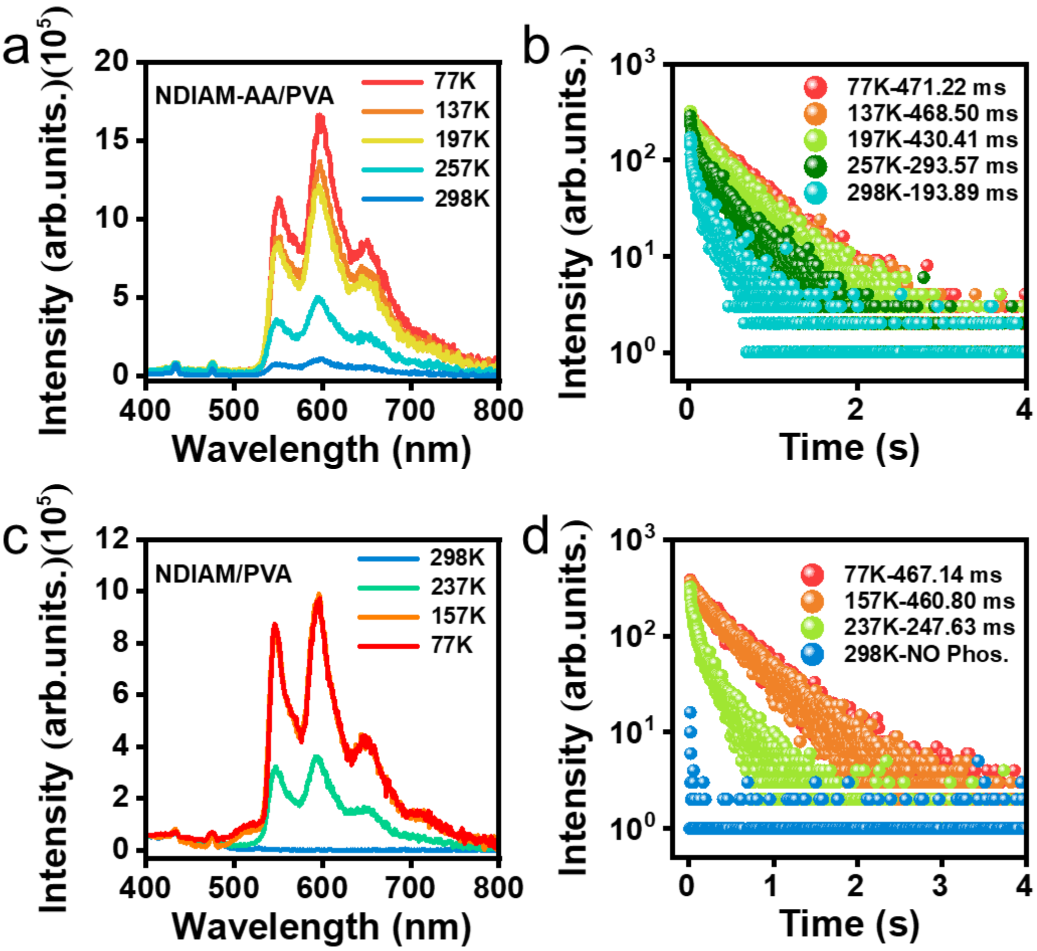

Supplementary Fig. 34 Temperature dependent phosphorescence spectra and lifetime of NDIAM-AA/PVA a) and b); NDIAM/PVA c) and d).

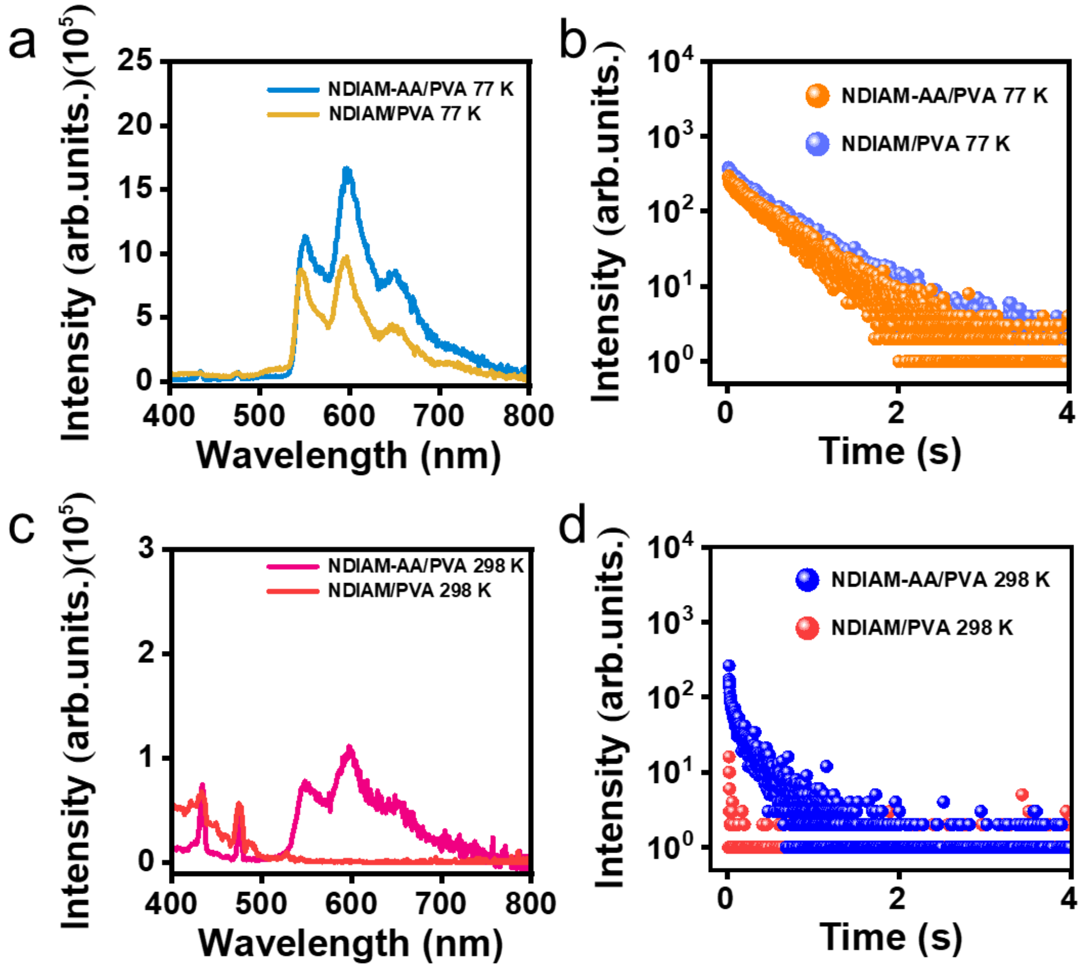

Supplementary Fig. 35 Phosphorescence intensity and Lifetime of NDIAM-PVA and NDIAM-AA/PVA at 77K a) and b); at 298K c) and d).

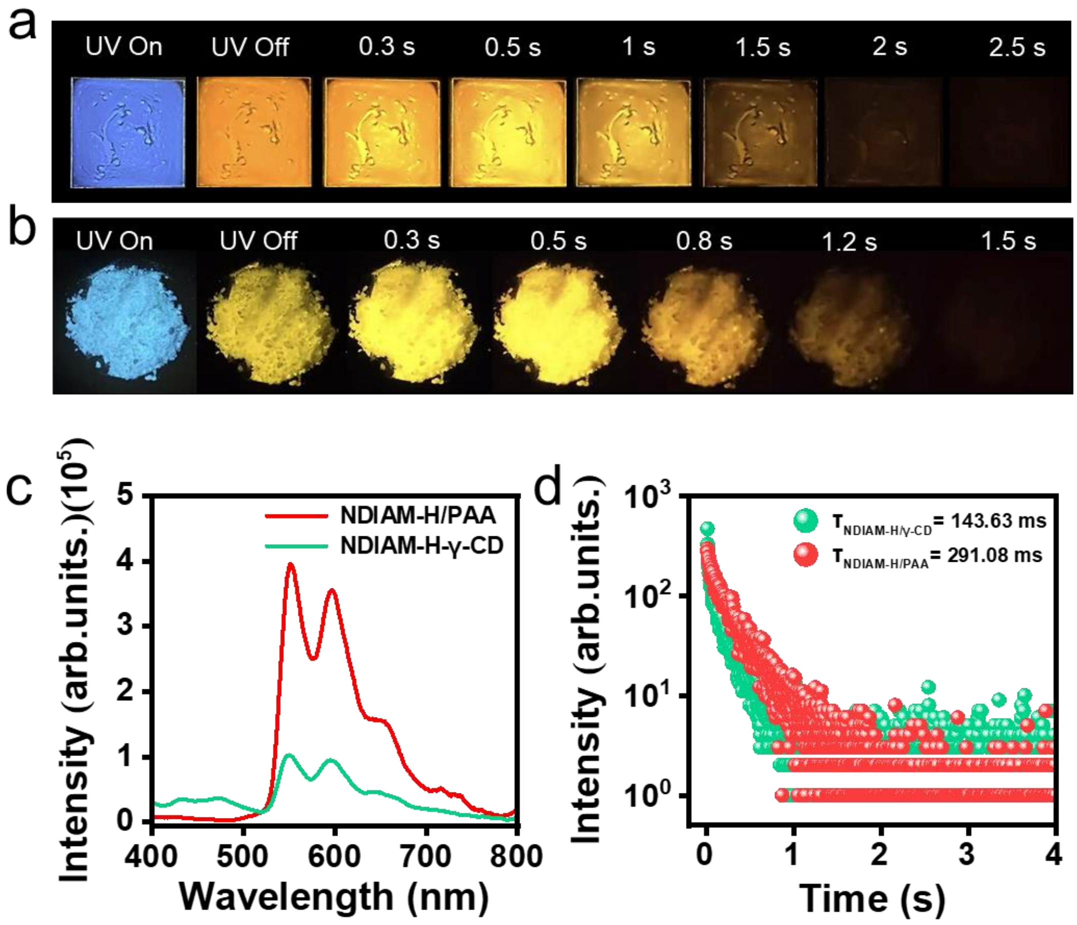

Supplementary Fig. 36 Photographs of a) NDIAM-H/PAA and b) NDIAM-H/γ-CD under and after ceasing 365 nm UV light irradiation; c) The delayed spectra of NDIAM-H/PAA and NDIAM-H/γ-CD; d) Phosphorescence lifetimes of NDIAM-H/PAA and NDIAM-H/γ-CD.

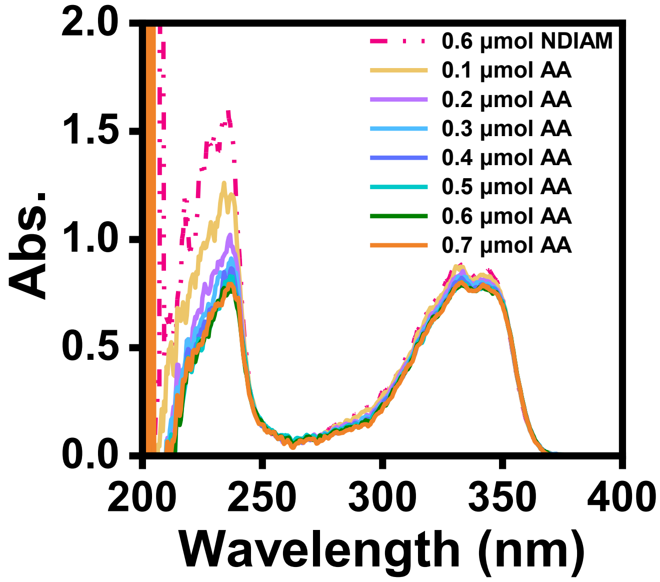

Supplementary Fig. 37 The absorption spectra at different molar weight of AA solution adding into NDIAM solution.

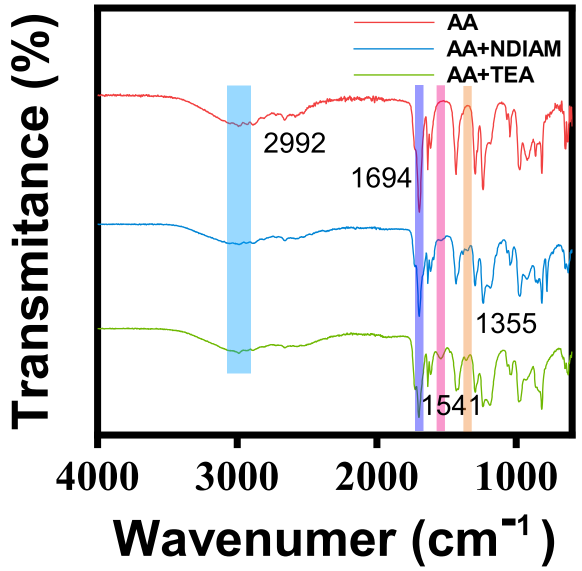

Supplementary Fig. 38 FTIR spectra of AA, AA-TEA and AA-NDIAM.

AA, NDIAM-AA (150 mg NDIAM and 600 μL AA), and triethylamine (TEA)-AA (200 μL TEA and 600 μL AA) of FTIR were also tested. Upon adding NDIAM and triethylamine (TEA) into AA solution, the vibration peaks of the hydroxyl at 2992 cm^{-1} and the carbonyl at 1694 cm^{-1} in carboxyl weaken; while the vibration

peaks of the asymmetric and symmetric the carboxylate at 1541 cm^{-1} and 1355 cm^{-1} appear.

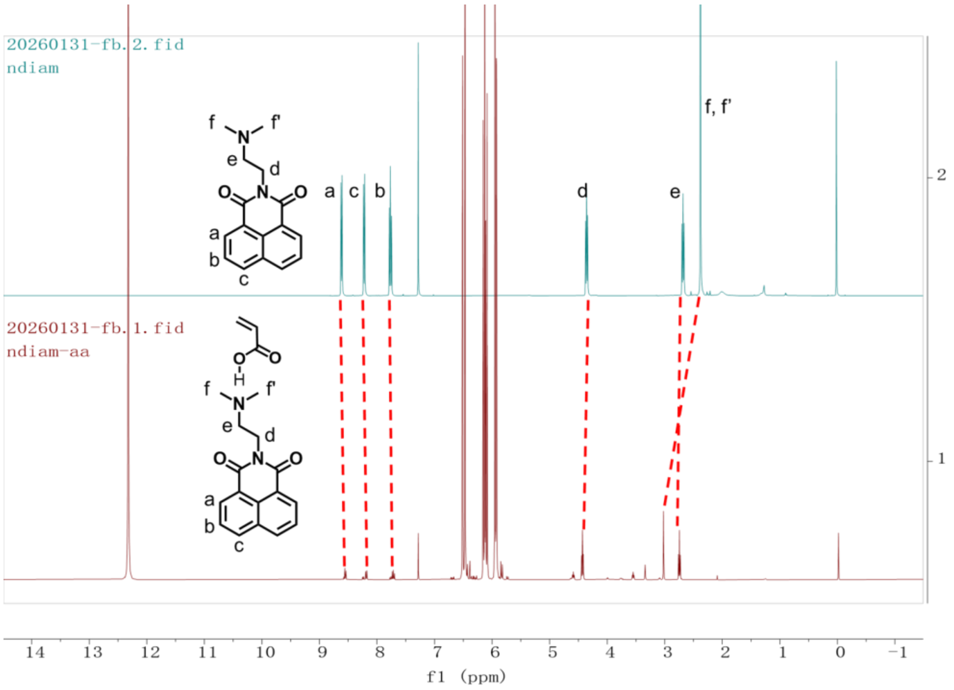

Supplementary Fig. 39 ^{1}H NMR (400 MHz, $CDCl_3$) of NDIAM and NDIAM-AA.

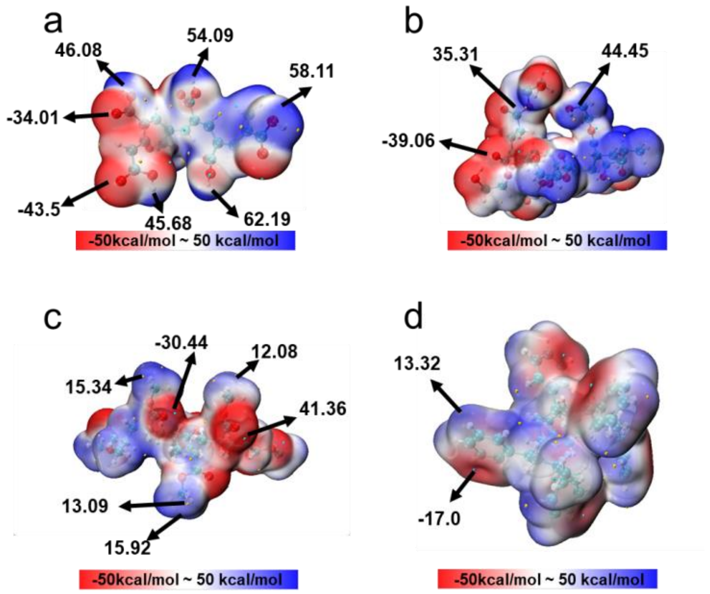

Supplementary Fig. 40 The electrostatic surface potential (ESP) analysis of a) PAA; b) PMA, c) PHEA and d) PSt.

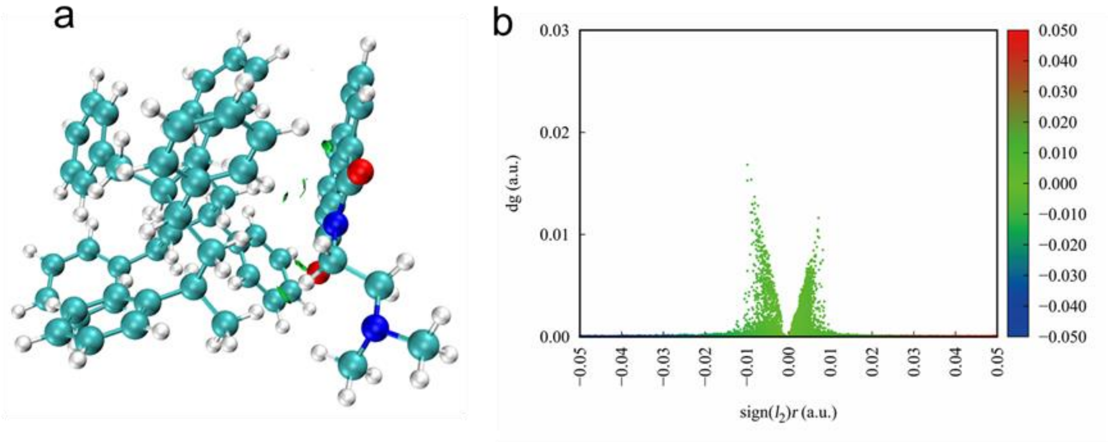

Supplementary Fig. 41 The independent gradient model based on Hirshfeld partition (IGMH) analysis of NDIAM/PSt.

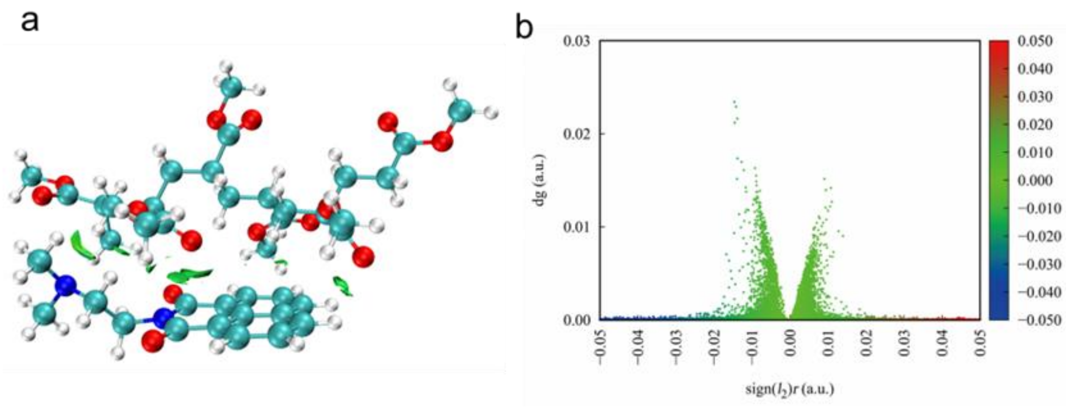

Supplementary Fig. 42 The independent gradient model based on Hirshfeld partition (IGMH) analysis of NDIAM/PMA.

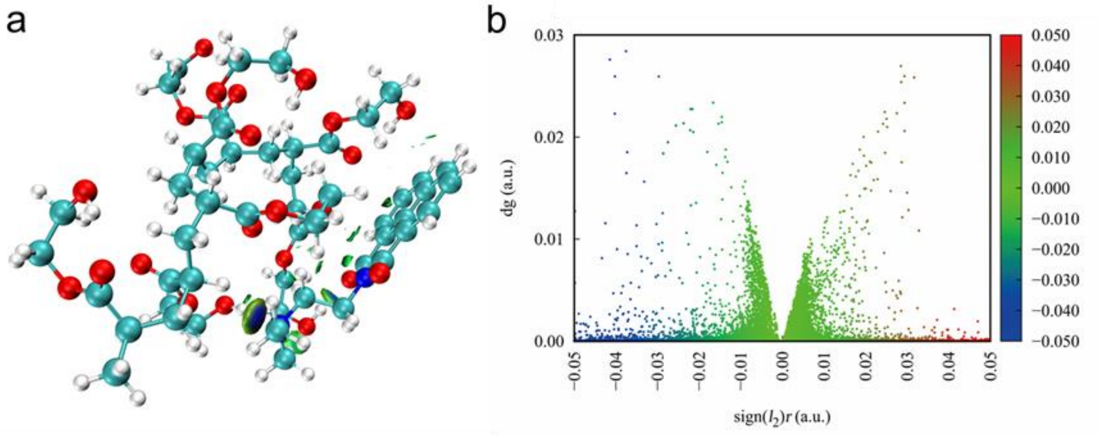

Supplementary Fig. 43 The independent gradient model based on Hirshfeld partition (IGMH) analysis of NDIAM/PHEA.

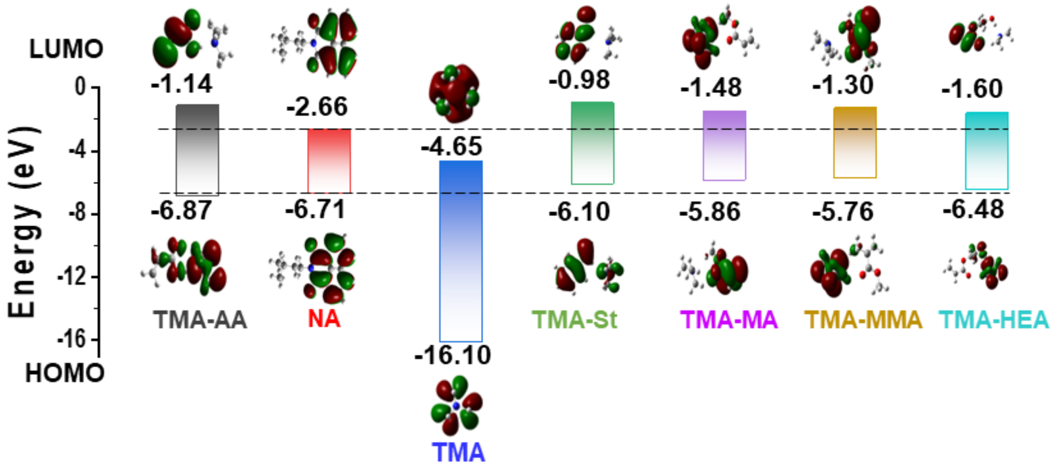

Supplementary Fig. 44 HOMO and LUMO of TMA-AA, NA, TMA, TMA-St, TMA-MA, TMA-MMA and TMA-HEA.

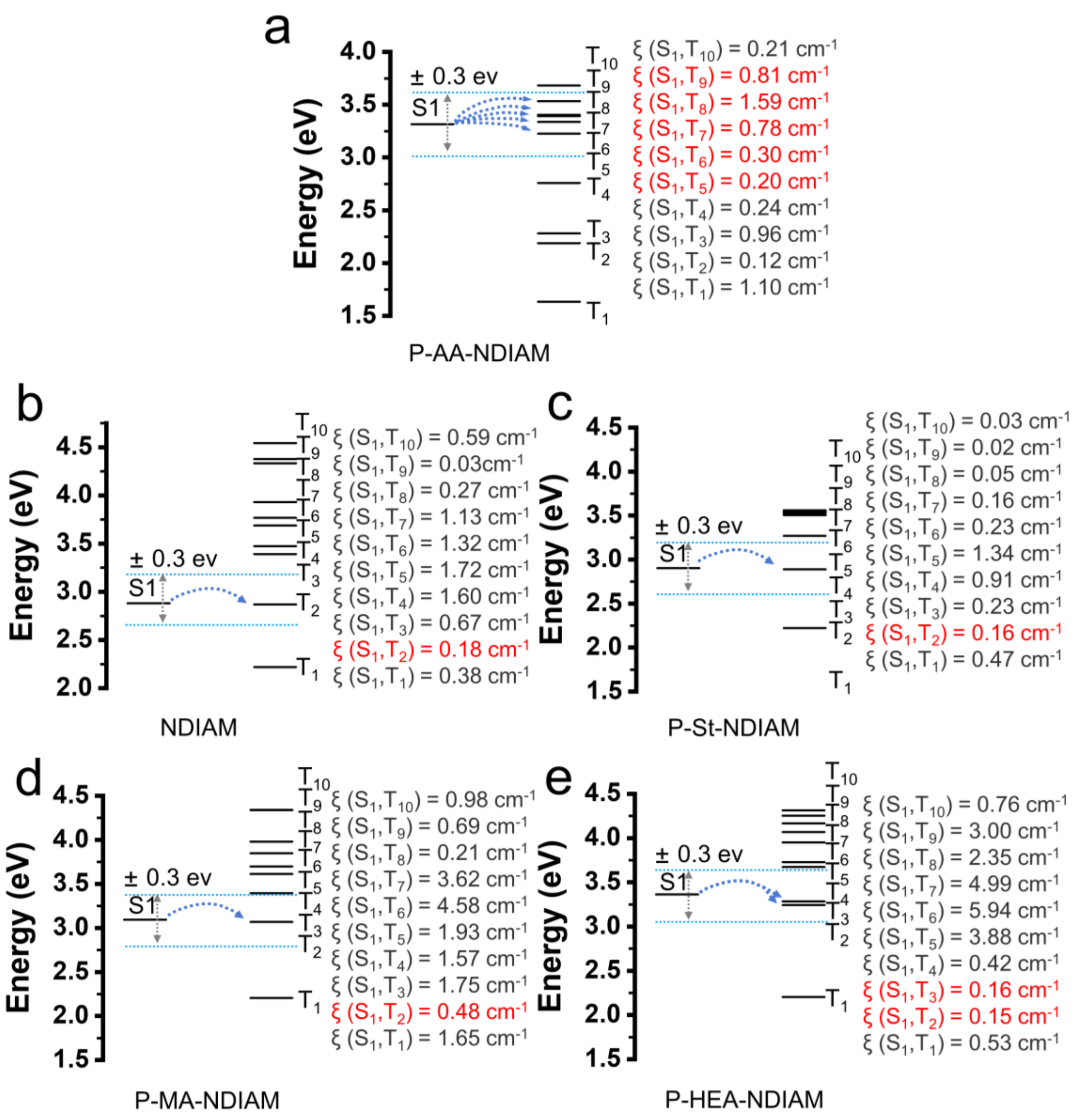

Supplementary Fig. 45 The energy levels and SOC constants of different photocuring RTP materials were calculated. a) P-AA-NDIAM; b) NDIAM; c) P-St-NDIAM; d) P-MA-NDIAM; e) P-HEA-NDIAM. SOC constants of S_1-T_n transitions available for ISC processes were highlighted in red.

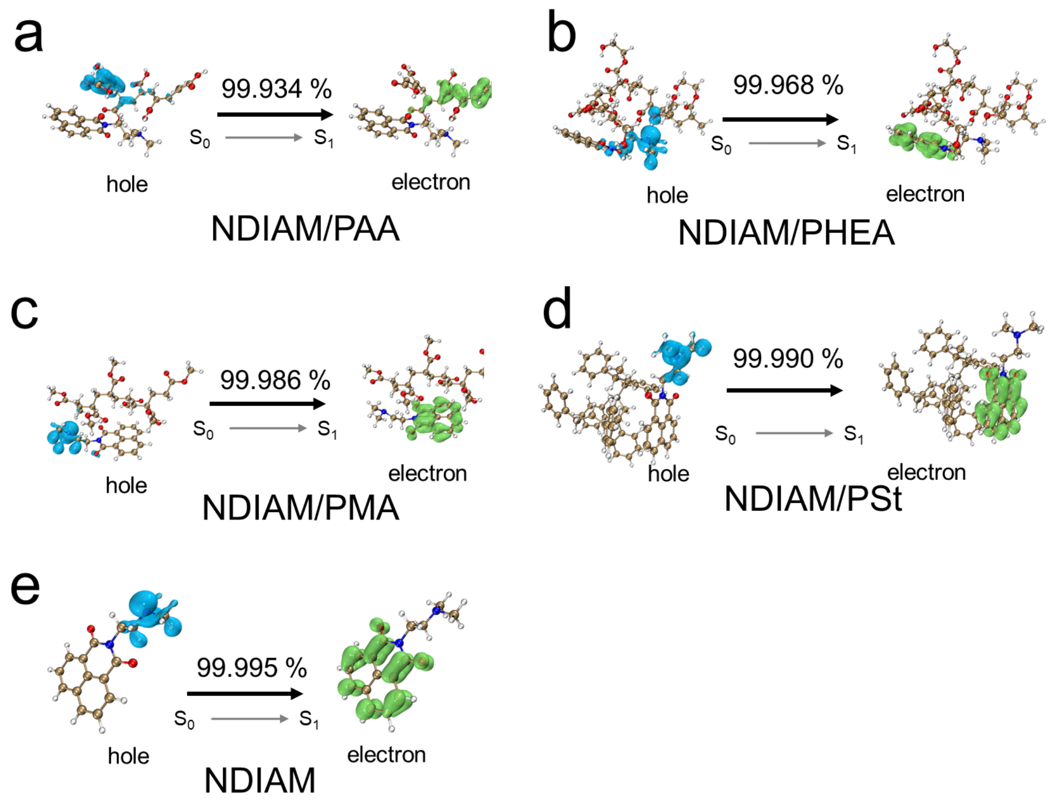

Supplementary Fig. 46 Hole and electron of S_1 state. a) NDIAM/PAA; b) NDIAM-PHEA; c) NDIAM/PMA; d) NDIAM-PSt; and e) NDIAM (green color: electron, blue color: hole).

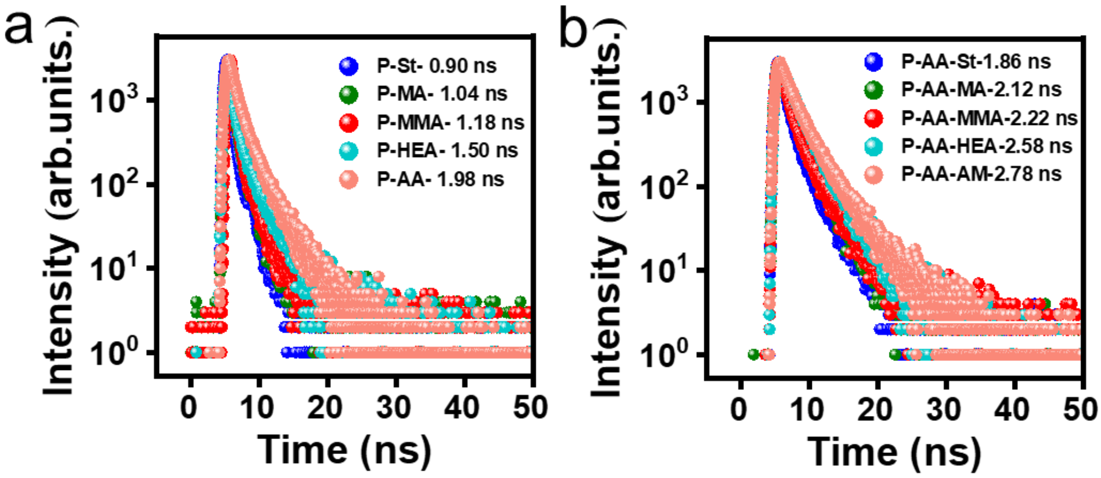

Supplementary Fig. 47 The fluorescence lifetime decay curves of different photocuring RTP materials.

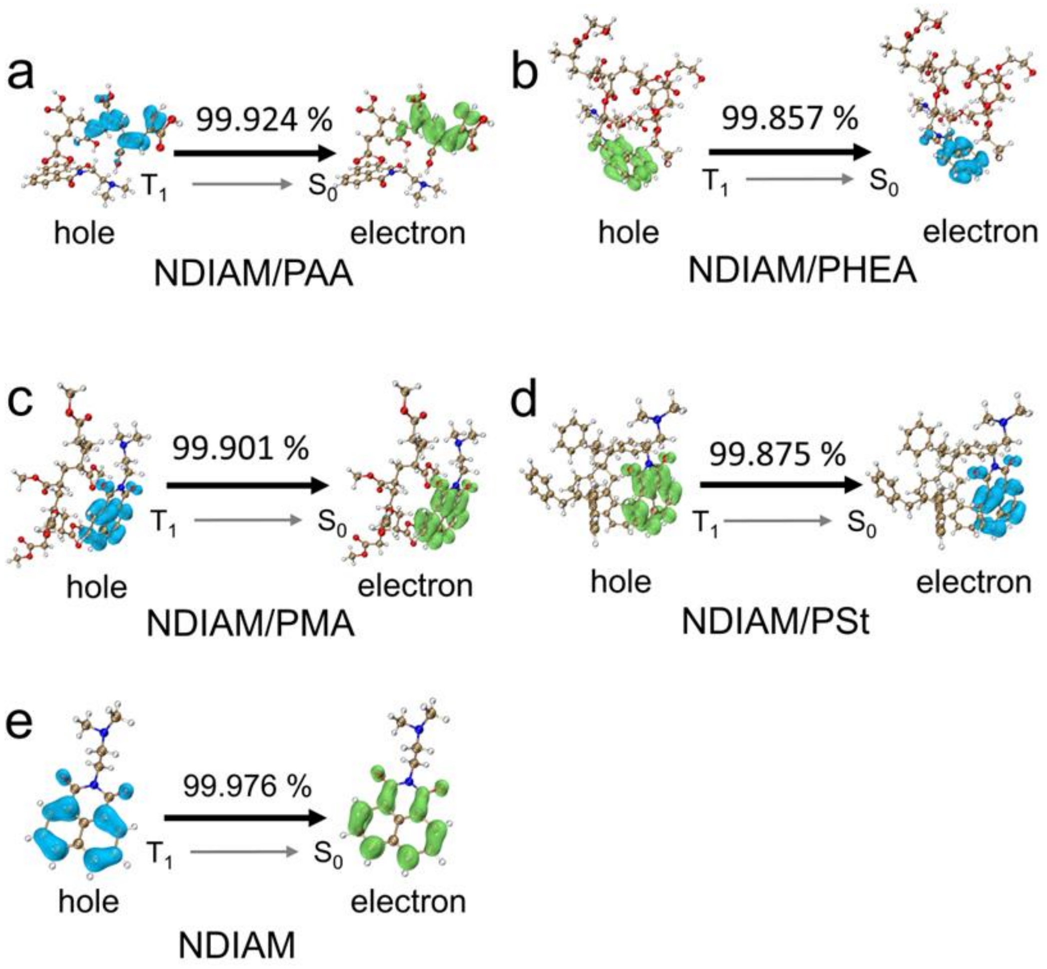

Supplementary Fig. 48 Hole and electron of T_1 state. a) NDIAM/PAA; b) NDIAM-PHEA; c) NDIAM/PMA; d) NDIAM-PSt; and e) NDIAM (blue color: hole, green color: electron).

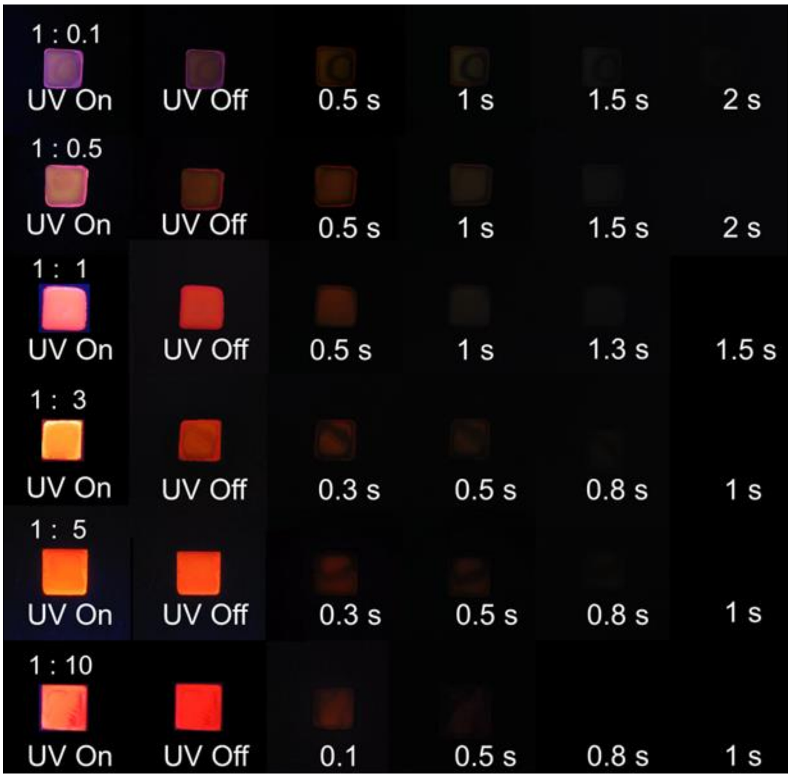

Supplementary Fig. 49 Afterglow images of the P-AA-AM/RhB photocured materials under 365 nm excitation. The NDIAM-to-RhB mass ratios are, from top to bottom, 1:0.1, 1:0.5, 1:1, 1:3, 1:5 and 1:10, respectively.

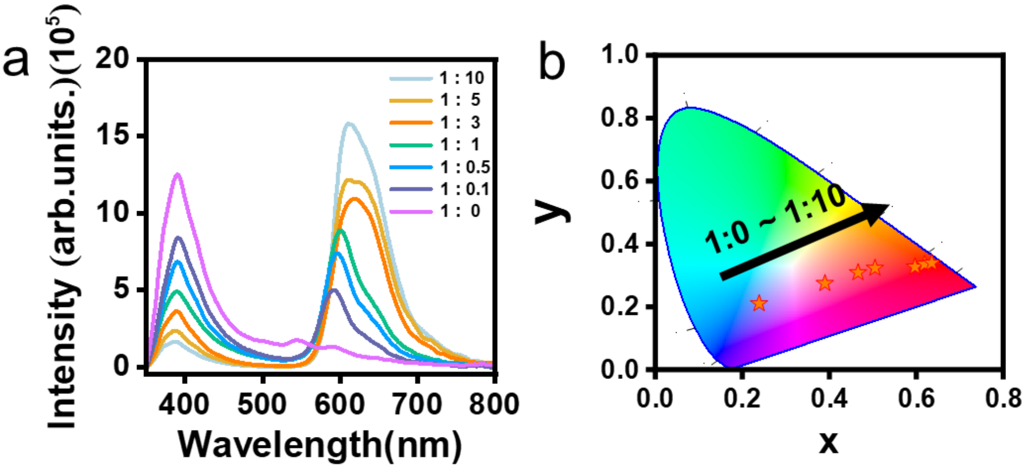

Supplementary Fig. 50 a) The fluorescence spectra of P-AA-AM/RhB with different RhB loadings. b) In the fluorescent state, CIE coordinates of P-AA-AM/RhB with different RhB contents.

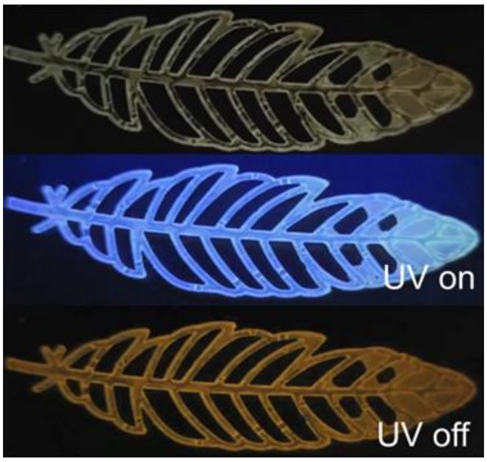

Supplementary Fig. 51 The images of 3D bulk materials made from P-AA-AM in the bright field, UV field and after removing the UV light source.

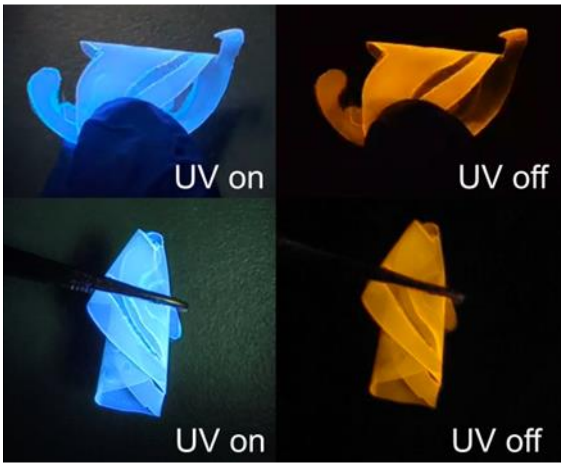

Supplementary Fig. 52 Photographs of the P-AA-AM photocured material under 365 nm UV illumination (left) and after removal of the UV source (right). The material is shown in a bent state (top) and a curled state (bottom).

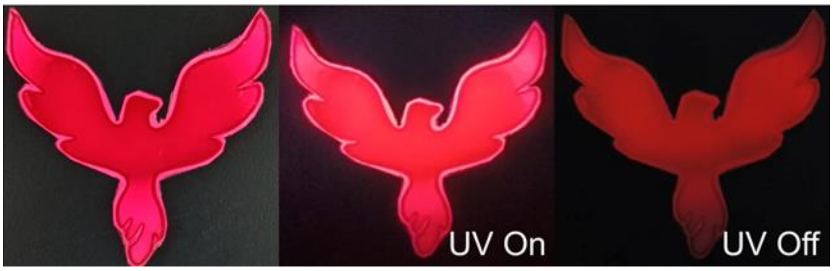

Supplementary Fig. 53 The afterglow picture of P-AA-AM/RhB photocuring material excited by 365 nm UV light source.

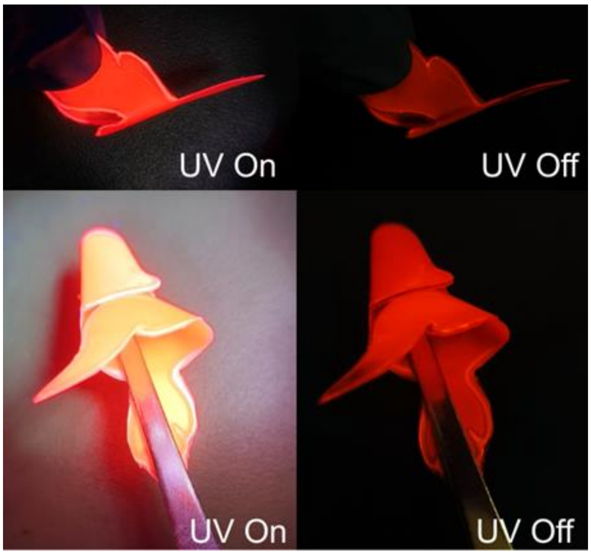

Supplementary Fig. 54 Photographs of the P-AA-AM/RhB photocured material under 365 nm UV illumination (left) and after removal of the UV source (right). The material is shown in a bent state (top) and a curled state (bottom).

Supplementary Table 1. HPLC quantitative analysis result of NDIAM methanol solution (10^{-4} M) elution for 15 min

Peak	Retention Time (min)	Area (Mau*s)	Height (Mau)	Area (%)
**1**	1.438	8952	294	0.078
**2**	2.260	6203	424	0.054
**3**	2.672	21967	1069	0.192
**4**	3.247	19446	964	0.170
**5**	3.450	2770	510	0.024
**6**	3.733	50270	3352	0.440
**7**	4.215	5146	659	0.045
**8**	5.263	11288435	786063	98.838
**9**	6.185	5842	561	0.051
**10**	7.476	9418	223	0.082
**11**	7.858	2673	165	0.023
**Totals**		11421123	794283	

Supplementary Table 2. Photophysical properties of RTP films

RTP materials	Φ_{PL} (%)	$\Phi_{Fluo.}$ (%)	$\Phi_{Phos.}$ (%)	$\tau_{Fluo.}$ (ns)	$\tau_{Phos.}$ (ms)
**P-AA-AM**	27.48	9.65	17.83	2.78	380.19
**P-AA-HEA**	11.15	3.44	7.71	2.58	248.26
**P-AA-MMA**	7.21	2.44	4.77	2.22	246.32
**P-AA-MA**	5.89	2.46	3.43	2.12	198.24
**P-AA-St**	4.13	1.98	2.15	1.86	81.9
**P-AA**	20.58	8.12	12.46	1.98	156.79
**P-HEA**	1.66	0.57	1.09	1.50	48.21
**P-MMA**	0.76	0.20	0.56	1.18	40.25
**P-MA**	0.06	0.09	0.51	1.04	21.45
**P-St**	0.98	0.11	0.87	0.90	23.26

Supplementary Table 3. Photophysical properties of RTP films

RTP materials	k_r^{Fluo} (×10^7 s^{-1})	k_{nr}^{Fluo} (×10^8 s^{-1})	k_{ISC} (×10^7 s^{-1})	k_r^{Phos} (s^{-1})	k_{nr}^{Phos} (s^{-1})
**P-AA-AM**	3.47	2.61	6.41	0.47	1.91
**P-AA-HEA**	1.33	3.44	2.99	0.31	3.58
**P-AA-MMA**	1.10	4.18	2.15	0.19	3.77
**P-AA-MA**	1.16	4.44	1.61	0.17	4.75
**P-AA-St**	1.06	5.15	1.16	0.26	11.71
**P-AA**	4.10	4.01	6.29	0.79	5.07
**P-HEA**	0.38	6.56	0.73	0.23	20.40
**P-MMA**	0.17	8.41	0.47	0.14	24.66
**P-MA**	0.87	9.61	0.49	0.24	46.59
**P-St**	0.12	1.10	0.97	0.37	42.57

Supplementary Table 4. Summary of the energy transfer efficiency (Φ_{FRET})

Acceptor	Donor (NDIAM) and Acceptor (Rh B) Ratio (w/w)	Average Lifetime (in ms) of NDIAM (λ_{exc}=365 nm)	Energy Transfer Efficiency (%)
**Rh B**	1:0	373.50	---
**Rh B**	1:0.1	318.04	14.8 %
**Rh B**	1:0.5	302.63	19.0 %
**Rh B**	1:1	241.58	35.3 %
**Rh B**	1:3	152.84	59.1 %
**Rh B**	1:5	119.45	68.0 %
**Rh B**	1:10	108.40	71.0 %

Supplementary Table 5. Transition energy of NDIAM and its different photocuring materials

	NDIAM		P-AA-NDIAM		P-HEA-NDIAM		P-MA-NDIAM		P-St-NDIAM	
n	S_n	T_n	S_n	T_n	S_n	T_n	S_n	T_n	S_n	T_n
1	2.882	2.220	3.316	1.635	3.362	2.202	3.093	2.205	2.903	2.221
2	3.805	2.869	3.336	2.189	3.731	3.243	3.778	3.069	3.513	2.89
3	3.874	3.392	3.538	2.281	3.964	3.285	3.943	3.394	3.554	3.271
4	4.140	3.474	3.56	2.759	4.055	3.673	4.131	3.614	3.708	3.506
5	4.240	3.687	3.725	3.227	4.063	3.729	4.334	3.699	3.732	3.524
6	4.511	3.767	3.894	3.337	4.179	3.949	4.535	3.848	3.793	3.537
7	4.587	3.932	3.928	3.393	4.391	4.061	4.603	3.981	3.824	3.541
8	4.613	4.355	3.949	3.405	4.431	4.167	4.668	4.337	3.827	3.549
9	5.157	4.377	3.983	3.534	4.488	4.254	4.751	4.341	3.846	3.56
10	5.488	4.543	4.027	3.682	4.605	4.313	4.768	4.57	3.912	3.563
