# Supplementary material for: Proton transfer regulated photocured robust room-temperature phosphorescence from naphthalimide
Source: Nat Commun. 2026 Mar 21;17:4287. doi: 10.1038/s41467-026-70999-8 (PMC13168491; doi:10.1038/s41467-026-70999-8)
Supplement: Supplementary file 2 — Description of Additional Supplementary Files [file 41467_2026_70999_MOESM2_ESM.pdf]

Description of Additional Supplementary Files

Supplementary Movie 1: The luminescence behavior of P-AA-AM before and after 365 nm UV light irradiation.

Supplementary Movie 2: The P-AA-AM / RhB precursor solution was placed in a mold for layer-by-layer curing molding to prepare a three-dimensional flower structure by layer-by-layer stacking photocuring 3D printing technology. The luminescence behavior of the 3D flower before and after 365 nm UV light irradiation.

Supplementary Movie 3: RTP material with butterfly pattern was prepared by photolithography. The luminescence behavior of butterfly pattern before and after 365 nm UV light irradiation.

Supplementary Movie 4: The luminescence behavior of P-AA-AM/RhB under dark conditions before and after 365 nm UV light irradiation.

Supplementary Movie 5: The RTP material of the school badge was prepared by photolithography. The luminescence behavior of the school badge before and after 365 nm UV light irradiation.

Supplementary Movie 6: The RTP material of the school two-dimensional code was prepared by photolithography. The luminescence behavior of the school two-dimensional code before and after 365 nm UV light irradiation.

Supplementary Movie 7: The luminescence behavior of the photocured yarn material coating with the P-AA-AM and P-AA-AM/RhB precursor solutions before and after 365 nm UV light irradiation.

Supplementary Data 1: Electronic structure calculations-atomic coordinates and molecular conformations.
